# Neural substrates of spatial processing and navigation in blindness: An activation likelihood estimation meta-analysis

**DOI:** 10.3389/fnins.2022.1010354

**Published:** 2022-10-20

**Authors:** Maxime Bleau, Samuel Paré, Daniel-Robert Chebat, Ron Kupers, Joseph Paul Nemargut, Maurice Ptito

**Affiliations:** ^1^École d’Optométrie, Université de Montréal, Montreal, QC, Canada; ^2^Visual and Cognitive Neuroscience Laboratory (VCN Lab), Department of Psychology, Faculty of Social Sciences and Humanities, Ariel University, Ariel, Israel; ^3^Navigation and Accessibility Research Center of Ariel University (NARCA), Ariel University, Ariel, Israel; ^4^Institute of Neuroscience, Faculty of Medicine, Université de Louvain, Brussels, Belgium; ^5^Department of Neuroscience, University of Copenhagen, Copenhagen, Denmark; ^6^Department of Neurology and Neurosurgery, Montreal Neurological Institute, McGill University, Montreal, QC, Canada

**Keywords:** visual impairments and blindness, spatial navigation, spatial processing, neuroplasticity, amodality, neuroimaging, MRI, meta-analysis

## Abstract

Even though vision is considered the best suited sensory modality to acquire spatial information, blind individuals can form spatial representations to navigate and orient themselves efficiently in space. Consequently, many studies support the *amodality hypothesis* of spatial representations since sensory modalities other than vision contribute to the formation of spatial representations, independently of visual experience and imagery. However, given the high variability in abilities and deficits observed in blind populations, a clear consensus about the neural representations of space has yet to be established. To this end, we performed a meta-analysis of the literature on the neural correlates of spatial processing and navigation via sensory modalities other than vision, like touch and audition, in individuals with early and late onset blindness. An activation likelihood estimation (ALE) analysis of the neuroimaging literature revealed that early blind individuals and sighted controls activate the same neural networks in the processing of non-visual spatial information and navigation, including the posterior parietal cortex, frontal eye fields, insula, and the hippocampal complex. Furthermore, blind individuals also recruit primary and associative occipital areas involved in visuo-spatial processing via cross-modal plasticity mechanisms. The scarcity of studies involving late blind individuals did not allow us to establish a clear consensus about the neural substrates of spatial representations in this specific population. In conclusion, the results of our analysis on neuroimaging studies involving early blind individuals support the *amodality hypothesis* of spatial representations.

## Introduction

Vision is the most adapted sense in humans for moving around and wayfinding (Ekstrom, 2015) and the most prominent spatio-cognitive sensory modality (Foulke, 1982). It has been hypothesized that all spatial inputs are recoded into *visual* representations in the brain (Pick, 1974; Lehnert and Zimmer, 2008). Consequently, blindness is often associated with an inability to move and orient oneself properly in space (Ptito et al., 2021a). However, through orientation and mobility (O&M) training, blind individuals learn to rely on other sensory modalities such as audition (i.e., echolocation), olfaction, touch and proprioception (Juurmaa and Suonio, 1975; Kupers et al., 2010; Long and Giudice, 2010; Teng et al., 2012; Kolarik et al., 2017) for resolving spatial tasks. Blind individuals also learn adaptive orientation strategies and the use of navigation and orientation aids, such as the long cane and tactile maps, which extend their perception of the environment and allow them to learn its layout (Long and Giudice, 2010; Chebat et al., 2018a,2020a; Giudice, 2018; Ptito et al., 2021a). This training thus allows safe and independent navigation for both early blind (EB) and late blind (LB) individuals (Long and Giudice, 2010; Schinazi et al., 2016). In fact, EB, even without visual experience, are able to form cognitive spatial representations (or cognitive maps) of various environments (Passini and Proulx, 1988; Passini et al., 1990; Thinus-Blanc and Gaunet, 1997; Fortin et al., 2006a; Connors et al., 2014; Chebat et al., 2018b; Nelson et al., 2018) through a process known as spatial learning, cognitive mapping or spatial knowledge acquisition (Schinazi et al., 2016; Chebat et al., 2020a). Hence, EB can integrate paths (Loomis et al., 1993, 2012), as well as encode and recognize routes and locations (Passini and Proulx, 1988; Passini et al., 1990; Thinus-Blanc and Gaunet, 1997; Fortin et al., 2006a).

Empirical data thus indicate that EB can represent space, and that their deficit is purely *perceptual*, and not *cognitive.* This conjecture is demonstrated by studies investigating the use of sensory substitution devices (SSDs) which translate visual information into other sensory modalities (i.e., vibrotactile or auditory feedback). These studies showed that when given the same amount of spatial information through SSDs, EB are able to locate and avoid obstacles as efficiently as LB and blindfolded sighted controls (SC), sometimes outperforming them (Segond et al., 2005; Auvray and Myin, 2009; Chebat et al., 2011; Maidenbaum et al., 2014; Stoll et al., 2015; Bleau et al., 2021; Paré et al., 2021) or performing as well as SC using vision (Chebat et al., 2015, 2017). Furthermore, EB, and even LB, outperformed blindfolded SC in various spatial tasks, such as non-visual path integration (Loomis et al., 1993) and locating sound sources on the horizontal plane (Voss et al., 2004; Doucet et al., 2005; Gougoux et al., 2005; Collignon et al., 2011). However, other studies suggests that EB exhibit impairments in auditory and proprioceptive spatial perception (Cappagli et al., 2017) and have deficits in many spatial tasks such as sound localization in the vertical plane (Zwiers et al., 2001; Lewald, 2002), auditory spatial bisection (Gori et al., 2014, 2020) and motion encoding (Finocchietti et al., 2015). Furthermore, other studies showed that even though EB can form and use spatial representations, they fail to achieve the same level of proficiency than LB and SC (Fortin et al., 2006b; Pasqualotto and Newell, 2007; Ruggiero et al., 2018, 2021). Hence, there is no consensus on the abilities and deficits of blind individuals, as they seem to vary depending on the chosen paradigms and testing conditions (Schinazi et al., 2016; Giudice, 2018).

It is however clear that blindness is associated with complex behavioral changes that vary with types of tasks, context, and different personal and social factors such as age, blindness onset, physical exercise and O&M training (Cappagli and Gori, 2016; Giudice, 2018; Rogge et al., 2021). The large variability in behavioral performances may thus be attributable to the wide range of adaptive strategies and abilities (e.g., Braille reading, echolocation) used by blind individuals to compensate for their lack of visual input. These life-long changes are also accompanied by underlying changes at the neural level (Merabet and Pascual-Leone, 2010; Kupers and Ptito, 2014). Indeed, it is now well known that the brain of blind individuals (EB and LB) undergoes important modifications both at the anatomical and functional levels. Occipital visual areas are cross-modally recruited by other sensory modalities through various neuroplastic mechanisms (Harrar et al., 2018; Chebat et al., 2020b; Ptito et al., 2021a). This cross-modal recruitment enables the visual cortex to stay functional and leads to better performances in non-visual tasks (Kupers and Ptito, 2014; Silva et al., 2018; Ptito et al., 2021a). However, this finding is at odds with reports of significant atrophy in visual pathways and occipital cortex (Shimony et al., 2006; Ptito et al., 2008b,2021b; Wang et al., 2013; Jiang et al., 2015). Furthermore, brain structures involved in navigation such as the posterior hippocampus, the parahippocampal gyrus and the entorhinal gyrus (Fyhn et al., 2007; Epstein et al., 2017), are reduced in EB, and to a certain extent in LB (Chebat et al., 2007; Ptito et al., 2008b; Modi et al., 2012; Ankeeta et al., 2021).

Studies conducted in blind individuals have led to the concept of an *amodal* foundation of the neural representation of space which has been conceptualized in the *amodality hypothesis* (Loomis et al., 2013). This hypothesis suggests that spatial representations can be formed through many different sensory inputs, are encoded in a format that transcends specific sensory modalities and are *spatial* in nature. According to this theory, all subsequent spatial mental operations are independent from their input modalities and from visual experience (Loomis et al., 2012; Chebat et al., 2018a; Giudice, 2018; Harrar et al., 2018). Thus, spatial deficits in the blind may not arise from the loss of vision *per se*, but from the insufficient access to spatial information due to the lack of non-visual alternatives and the poor accessibility of the environment built by and for the sighted. Consequently, cumulating and integrating spatial information for spatial knowledge acquisition is a longer and more cognitively demanding process that heavily depends on the individual’s attentional resources and memory (Long and Giudice, 2010; Giudice, 2018). This may lead to delays in the development of spatial abilities in the blind who rely more frequently on egocentric frames of reference (based on the subject’s viewpoint) instead of allocentric ones (independent from the individual’s perspective). Blind individuals therefore often prefer route-based strategies rather than cognitive map-based, or “survey knowledge,” strategies that are more prone to error in this population (Passini and Proulx, 1988; Millar, 1994; Thinus-Blanc and Gaunet, 1997; Ungar et al., 1997; Espinosa et al., 1998; Pasqualotto and Proulx, 2012; Iachini et al., 2014; Giudice, 2018; Ruggiero et al., 2021).

As highlighted in the previous paragraphs, there is still no clear consensus regarding the nature of spatial processing and its independence from visual experience and imagery (Giudice, 2018). To the best of our knowledge, only two meta-analyses investigated neural correlates of non-visual functions in individuals with blindness (Ricciardi et al., 2014; Zhang et al., 2019). These studies however mainly focused on the cross-modal recruitment of the occipital cortex and not on the functioning of the larger neural network underlying spatial navigation in the absence of vision (Chebat et al., 2018a,2020b). Furthermore, results from LB were excluded. Therefore, it is relevant and timely to conduct a meta-analysis specifically on the neural correlates of non-visual spatial navigation and orientation in both EB and LB. This type of analysis may help to shed light on potential knowledge gaps and lead to new research directions in the field of O&M, visual rehabilitation and restoration. The present study hence examines brain activations patterns in a large data set of spatio-cognitive paradigms in EB, LB, and SC. The systematic literature search focuses on paradigms investigating cognitive processes involved in (1) the processing of spatial information through tactile and auditory modalities; and (2) spatial navigation and orientation in the absence of vision. Activation likelihood estimation (ALE) analyses were performed to investigate general agreements across the selected neuroimaging studies. Results identified shared activations in frontoparietal networks involved in visuospatial attention, and recruitment of navigation networks (e.g., hippocampus, parahippocampus, and other areas of the visual dorsal stream) in EB, as compared to LB and SC. Current data thus support the view that spatial representations in these networks are indeed independent from the input modality.

## Methods

The literature search conducted in the present study as well as the screening and selection process followed the *Preferred reporting items for systematic reviews and meta-analyses* (PRISMA) guidelines (Moher et al., 2009). We systematically searched seven databases: PubMed (NCBI), PsycINFO (EBSCO), MEDLINE (NLM), Global Health (PSI), Embase (Elsevier), ERIC (IES), and Web of Science (Clarivate Analytics), to identify neuroimaging studies that investigated spatial navigation or spatial processing in individuals with total blindness, and that were published between January 1990 and January 2022. Since spatial navigation tasks generally integrate different spatial and decision-making tasks, data in the selected articles were extracted and divided into two categories: (1) Non-visual *Spatial Processing* (tasks generally involving one specific type of spatial computing) and (2) Non-visual *Spatial Navigation* (tasks involving higher order processes, i.e., navigating, recognizing routes or important features used during wayfinding). Following this classification, ALE meta-analyses were performed to identify the neural correlates in EB, LB and SC for these two categories. Contrast and conjunction meta-analyses were also conducted to identify the neural correlates that are common, or different, across the three groups. In the context of this paper, we defined EB as individuals who either were born blind or acquired blindness early in life (<1 year of age), whereas individuals who acquired blindness later in life were considered as LB.

### Literature search

The *population* of interest was defined as adult (≥18 years of age) individuals with total blindness resulting from peripheral (eye or optic nerve) pathologies, or damages, without neurological disorders or psychiatric illness. The topic of interest was related to the neural substrates of spatial processing, spatial navigation, and orientation, using sensory information other than vision (i.e., tactile and auditory). In the meta-analysis, we considered neuroimaging studies published before January 2022, and written in English. Thereupon, a broad list of keywords relating to “spatial navigation” or “spatial processing” and “neural correlates” and “blindness” was then developed to collect relevant articles (Supplementary Table 1). In addition, through snowballing (Jalali and Wohlin, 2012), reference lists of the most relevant empirical articles and reviews were also screened to identify any other eligible articles. All identified sources were imported into End-Note v.9.3.3 (Clarivate Analytics, PA, USA) and exported into Covidence (Veritas Health Innovation, Melbourne, Australia), a screening software for the conduction of systematic reviews. All duplicates were then automatically removed, and a two-stage screening process was conducted: (1) title and abstract screening; and (2) full-text screening. This procedure allowed for the identification of eligible articles based on the inclusion criteria and *post hoc* exclusion criteria (Table 1). During title and abstract screening, only articles including blind participants, investigating spatial navigation or spatial sensory processing, and employing task-based fMRI or PET techniques were withheld. During the full-text screening process, we followed guidelines for conducting meta-analysis (Müller et al., 2018) and only accepted papers that (1) reported activation coordinates in standardized Talairach space (Talairach and Tournoux, 1988) or Montreal Neurological Institute (MNI) space (Collins et al., 1994); (2) conducted within-group based contrasts; (3) performed whole-brain analyses; (4) used an univariate approach to reveal localized increased activations; and (5) included a minimum of three participants per group in the final analyses. The final criterion was modified due to recruitment challenges often encountered when working with blind populations (see “Study limitations and considerations for future research” section).

**TABLE 1:** Inclusion and exclusion criteria.

Inclusion criteria	Exclusion criteria
• Studies on non-visual spatial navigation or spatial sensory processing. • Studies involving individuals with total blindness (bilateral & peripheral). • Neuroimaging methods of interest: only fMRI and PET.	***According to Participants, Concept and Context*** • Presence of concomitant neurological disorders or psychiatric illnesses. • Spatial tasks not relevant to spatial navigation or with no sufficient control (i.e., imagery with no sensory stimuli/task, etc.). ***According to Meta-analysis guidelines*** • *MNI* or *Talairach* coordinates not reported. • No univariate analysis (i.e., multivoxel analysis, machine learning, functional connectivity). • No “within group”- based contrasts. • Region of Interest (ROI) instead of whole brain analysis. • Less than three participants per group.

Ultimately, 31 studies were included in the meta-analysis that were grouped into two categories: (1) *spatial processing* (21 studies), including articles with spatial tasks (i.e., localization, spatial attention, spatial working memory), that required the processing of non-visual sensory stimuli (of these, 13 studies investigated the auditory modality and seven, the tactile modality, while only one investigated both); and (2) *spatial navigation* (9 studies), including articles with spatial navigation tasks in a given environment or spatial tasks that required the processing of important information used by individuals with blindness during navigation and spatial learning (i.e., spatial language, landmarks or spatially significant textures).

**FIGURE 1:**
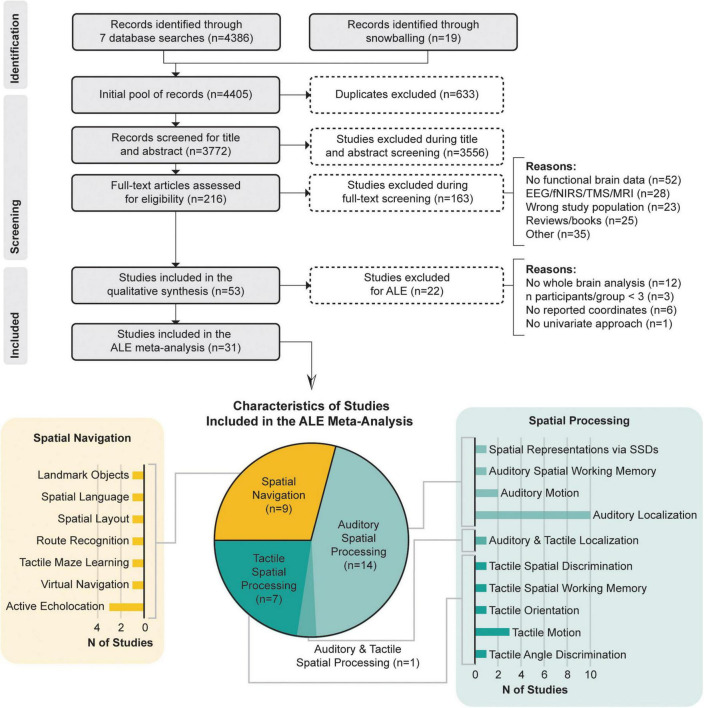
PRISMA flow chart and characteristics of studies included in the meta-analysis according to datasets and the specific functional domains they investigated.

Figure 1 illustrates the literature search process and the classification of the included papers.

Data from every included article was extracted and organized based on authors’ names, year of publication, characteristics of participants, neuroimaging methodology (fMRI or PET), contrasts of interest (only those reflecting spatial processes), coordinates space (MNI or Talairach), number of activation foci identified for the performed contrasts, and significant finding related to the aim of the present study. Table 2 presents a detailed overlook of the 31 studies included in the meta-analysis, including to which category dataset articles belonged and the functional domain of the task (in other words, the nature of the task or of the investigated spatial process). Since four out of 31 studies (Gougoux et al., 2005; Voss et al., 2006, 2008, 2011) performed the same experiments in three independent samples (EB, LB, and SC), included articles may feature potential overlaps of subject samples. Information about sources that were not included in the meta-analysis is provided in Supplementary Table 2.

**TABLE 2:** Overview of included studies.

REFERENCES	FUNCTIONAL DOMAIN	METHOD	*N* SUBJECTS	TASK CONTRASTS	FOCI EB	FOCI LB	FOCI SC	MAIN FINDINGS
***SPATIAL NAVIGATION***								
Dodsworth, 2019 *	Active echolocation	fMRI	5 Blind (4 EB, 1 LB), 8 SC	Active echolocation for navigation, with echoes > No echoes; Route > Scrambled route	30	–	19	During echolocation-based navigation, expert echolocators recruited parts of the occipital cortex (MOG, cuneus, precuneus) and small area in the parahippocampus.
Fiehler et al., 2015	Active echolocation	fMRI	6 EB, 3 SC	[expert or novice] Active echolocation for path direction, with echoes > No echoes	6	–	9	Path direction discrimination via echoes recruited premotor cortex, SPL, IPL and IFC in all groups. EB additionally recruited parts of the occipital cortex.
Gagnon et al., 2012	Tactile maze learning	fMRI	11 EB, 14 SC	Maze learning > Rest	16	–	5	EB activated the right hippocampus and parahippocampus, occipital cortex and fusiform gyrus. Those activations increased across three runs.
Halko et al., 2014	Virtual navigation	fMRI	9 EB	Virtual navigation > Motor control; Planning period > Motor control	14	–	–	Right TPJ activation during planning and execution of the task, which correlated with performance and subjective independence level in daily travels.
He et al., 2013	Landmark objects	fMRI	16 EB, 17 SC	Large non-manipulable objects > tools & animals	5	–	5	EB and SC activate PPA, RSC and left TOS in both visual (pictures of objects and scenes) and auditory (language) modalities.
Kupers et al., 2010	SSD & route recognition	fMRI	10 EB, 10 SC	Route recognition > Scrambled route	30	–	20	Like SC (full Vision), EB activated areas in the visual cortex (IOG, SOG, MOG, cuneus and fusiform gyrus), right parahippocampus, superior and inferior PPC, precuneus, anterior cingulate cortex, anterior insula, dorsolateral PFC, and cerebellum.
Milne et al., 2015	Active echolocation	fMRI	6 EB, 3 SC	[expert or novice] Active echolocation, with material echoes > No echoes	20	–	6	EB expert echolocators activated calcarine sulcus during echolocation and parahippocampal cortex (parahippocampal gyrus, fusiform gyrus, and anterior CoS) to identify surface material through echoes. No parahippocampal, nor occipital responses were found in EB novice echolocators and SC.
Struiksma et al., 2011	Spatial language	fMRI	13 EB, 13 SC	Spatial > Non-spatial sentences	24	–	24	EB activated left SMG, left MOG and right cuneus when spatial sentences were presented
Wolbers et al., 2011 *	Spatial layout	fMRI	4 EB, 3 LB, 7 SC	Spatial Layout > Non-spatial object	12	12	5	For spatial layouts, EB activated PPA, RSC, parieto-occipital sulcus, SPL area 7p and middle frontal gyrus. Compared to SC, EB had stronger activation of occipital and middle temporal areas.
***SPATIAL PROCESSING***								
Anurova et al., 2015	Auditory localization	fMRI	12 EB, 12 SC	Auditory identification and localization > Detection	25	–	13	In the auditory localization task, EB displayed higher (compared to SC) activations in regions of the occipital cortex, in the bilateral SPL, and in the left middle frontal gyrus and sulcus.
Bedny et al., 2010	Auditory motion processing	fMRI	10 EB, 5 LB, 21 SC	High motion + low motion > Rest	3	0	9	hMT/hMST response only found in EB.
Bonino et al., 2008	Tactile spatial working memory	fMRI	4 EB	Tactile recognition and spatial memory maintenance > Rest	76	–	–	EB activate (pre)frontal (premotor, SMA, PFC) and parietal cortical (SPL, precuneus, IPS) areas, as well as lateral occipital cortex and cerebellum.
Bonino et al., 2015	Tactile angle discrimination	fMRI	9 EB, 10 SC	Angle discrimination > Control	3	–	9	EB and SC recruited PPC, intraparietal regions, middle frontal and premotor areas. EB also recruited ventro-temporal, temporo-occipital and dorsal occipital regions. These activations were correlated to behavioral performance.
Chan et al., 2012	Auditory localization	fMRI	11 EB, 14 SC	Distance estimation > Detection	18	–	16	After training, EB increasingly activated R-inferior parietal cortex, L-hippocampus, R-cuneus, whereas no difference was found for SC in pre *vs* post-training.
Collignon et al., 2011	Auditory localization	fMRI	11 EB, 11 SC	Spatial judgment > Pitch judgment	36	–	14	Spatial judgments involved the dorsal network (IPL, SPL, middle occipito-temporal gyrus, etc.). EB showed higher activations in occipital regions known for visuospatial processing (cuneus, MOG, lingual gyrus).
Dormal et al., 2016	Auditory motion processing	fMRI	16 EB, 15 SC	Motion perception > Static	9	–	10	Auditory motion activated fronto-temporo-parietal network in both groups; only EB activated occipital areas (bilateral MOG and SOG), including areas hMT + /V5 and V3A.
Gougoux et al., 2005	Auditory localization	PET	12 EB, 7 SC	[Superior or Normal] Binaural and Monaural Sound Localization > Control	36	–	24	Both groups activated inferior parietal cortex. Occipital activations (striate and ventral extrastriate cortex) only in EB with superior sound localization. More extensive frontal activations for monaural localization.
Matteau et al., 2010	Tactile motion processing	fMRI	8 EB, 9 SC	Motion perception > Rest	24	–	17	hMT +, parietal (IPL, SPL) and frontal activations in EB and SC. EB also recruited right occipital cortex (BA 19).
Park et al., 2011	Auditory spatial working memory	fMRI	10 EB, 10 SC	2-back Working Memory of Location > 0-back Detection	11	–	7	Both groups activated prefrontal and parietal regions, and cerebellum. Fusiform gyrus and visual occipital areas (MOG, lingual gyrus, cuneus) showed increased functional connectivity with frontoparietal regions and default mode network in both groups.
Ptito and Kupers, 2005	SSD & tactile orientation processing	PET	6 EB, 5 SC	Pattern Orientation Detection > Random dots (no pattern)	19	–	18	After training with TDU, only EB activated occipital (cuneus, inferior, medial and lateral occipital cortex), occipito-parietal (dorsal IPS) and occipito-temporal (fusiform gyrus) areas. EB and SC activated frontal (IFG, SFG, MedFG, Insula) and parietal (anterior IPS) areas.
Ptito et al., 2009	Tactile motion processing	PET	7 EB, 6 SC	Motion Discrimination > Rest	10	–	10	EB recruited hMT, V1 (cuneus), V3 (MOG), IPS. EB and SC activated frontal areas (MFG, MedFG), SMG and cerebellum. SC activated PCG and insula.
Renier et al., 2010	Auditory and tactile localization	fMRI	12 EB, 12 SC	Localization > Identification or Detection	26	–	17	R-MOG activity during auditory and tactile spatial processing in EB; this activity was correlated with localization performance. R-MOG deactivation in blindfolded SC.
Ricciardi et al., 2007	Tactile motion processing	fMRI	4 EB, 7 SC	Motion Stimuli > Static Stimuli	38	–	25	EB and SC activated hMT +, parietal (IPS), and ventral and inferior temporal regions. Only EB activated motion-responsive areas V3A and V7.
Stilla et al., 2008	Tactile spatial processing	fMRI	5 EB, 5 LB	Tactile microspatial discrimination > Tactile temporal discrimination	41	20	–	Somatosensory, posterior and ventral IPS, frontal (FEF, PMv) and occipital (IOS, LOC, fusiform gyrus) areas were spatially responsive in both EB and LB.
Striem-Amit et al., 2012	SSD & spatial representations	fMRI	11 EB, 9 SC	Location > Shape	14	–	15	In both EB and SC, the location task activated parts of the dorsal stream, including the precuneus. Only EB activated occipital visual areas, including V1.
Tao et al., 2015	Auditory localization	fMRI	15 EB, 17 LB	Localization > differentiation	11	4	–	EB more strongly activate occipital areas (R-MOG), while LB more strongly activated prefrontal areas involved in visuospatial working memory.
Tao et al., 2017	Auditory localization	fMRI	11 EB, 13 LB	Localization > discrimination	7	7	–	After sound localization training, precuneus activation decreased in EB and increased in LB. In LB, visuospatial working memory capacities were linked to a precuneus-lingual gyrus network and enhanced learning of sound localization. The precuneus seems important to learn sound localization, independently from visual experience.
Voss et al., 2006	Auditory localization	PET	6 LB, 7 SC	Binaural and monaural sound localization > Control	–	7	0	LB did not demonstrate enhanced performances compared to SC, but showed occipital activations (in MOG, SOG, and lingual gyrus) during sound localization tasks. These activations were mostly in the right hemisphere during binaural localization but extended bilaterally in monaural localization. SC showed occipital deactivations. Parietal and frontal activations were also present.
Voss et al., 2008	Auditory localization	PET	12 EB, 6 LB, 7 SC	Binaural and monaural sound localization > Control	19	16	7	EB, but not LB, activated occipital visual areas. LB activated right medial and lateral occipitotemporal cortex.
Voss et al., 2011	Auditory localization	PET	12 EB, 6 LB, 7 SC	[Supperior or normal] Monaural sound localization with spectral cues > Control	15	19	14	Occipital activations (cuneus, lingual gyrus, IOG) in all groups but stronger in EB and LB. Activations in left lingual gyrus, left precentral sulcus and inferior frontal cortex boundary correlated with performance.
Weeks et al., 2000	Auditory localization	PET	9 EB, 9 SC	Localization > Rest	17	–	12	EB activated occipital cortex (parieto-occipital, dorsal, ventral). Both EB and SC activated IPL. Correlations between right IPL, temporal and occipital cortex (parieto-occipital, ventral, dorsal and peristriate) activations in EB.

*Studies that included a blind group with mixed EB and LB participants. CoS, collateral sulcus; FEF, frontal eye fields; IFC, Inferior frontal cortex; IOG, inferior occipital gyrus; IOS, intra-occipital sulcus; IPL, inferior parietal lobule; IPS, intraparietal sulcus; LOC, lateral occipital cortex; MedFG, medial frontal gyrus; MFG, middle frontal gyrus; MOG, middle occipital gyrus; hMST, human medial superior temporal area; hMT, human middle temporal area; MTG, middle temporal Gyrus; PFC, prefrontal cortex; PMv, ventral premotor cortex; PPA, parahipocampal place area; ROI, region of interest; RSC, retrosplenial complex; SMA, supplementary motor area; SMG, supramarginal gyrus; SOG, superior occipital gyrus; SSD, sensory substitution device; TDU, tongue display unit; TOS, transverse occipital sulcus; TPJ, temporal parietal junction.

### Datasets associated with non-visual spatial processing and navigation

The meta-analysis included a total of 263 EB, 50 LB and 232 SC, from 30, 9 and 26 studies, respectively. Reported coordinates of foci from all 31 included studies were extracted into seven datasets. For experiments in the *spatial processing* category, two datasets were constructed: “*EB Spatial Processing*” (20 contrasts, 461 foci; 183 subjects) and “*SC Spatial Processing*” (15 contrasts, 237 foci; 157 subjects). For the *spatial navigation* category, two datasets were constructed: “*EB Spatial Navigation*” (11 contrasts, 157 foci; 80 subjects) and “*SC Spatial Navigation*” (8 contrasts, 93 foci; 75 subjects). Furthermore, three datasets combining both spatial processing and navigation were formed: “*EB Spatial Processing* + *Spatial Navigation*” (31 contrasts, 618 foci; 263 subjects); “*SC Spatial Processing* + *Spatial Navigation*” (23 contrasts, 330 foci; 232 subjects); and “*LB Spatial Processing* + *Spatial Navigation.*” Since only a limited number of studies involved LB, the “*LB Spatial Processing* + *Spatial Navigation*” dataset only included 6 contrasts (78 foci; 50 subjects) which is well below guidelines criteria (Müller et al., 2018). Therefore, a meta-analysis specific to LB is still unachievable as of the current literature. In constructing these datasets, clusters with positive activations were included, while deactivation clusters were excluded. Different contrasts obtained from the same sample within the same article and/or across multiple articles (Gougoux et al., 2005; Voss et al., 2006, 2008, 2011) were pooled into one experiment to avoid counting a single experiment multiple time (Müller et al., 2018).

### Meta-analysis

We used the *Ginger*ALE software (version 3.0)^1^ to perform the meta-analysis. First, we converted foci from Talairach coordinates into MNI space. Then, three types of meta-analysis were conducted to determine the neural correlates of non-visual spatial navigation and spatial processing. Single meta-analyses (Eickhoff et al., 2012) were conducted on each dataset independently, to discover convergent clusters across tasks and participants. Contrast meta-analyses (Eickhoff et al., 2011) were conducted to detect clusters with significantly stronger activations (BOLD signal) in one group compared to the other. Contrast meta-analyses were performed to determine group differences in brain activations. The contrasts of interest were EB > SC, SC > EB. Conjunction meta-analyses (Eickhoff et al., 2011) were conducted to detect overlapping activated brain regions between groups. The specific investigated conjunctions were EB∩SC.

### Statistical analysis

For the single meta-analyses, an ALE value was calculated for each voxel, using cluster-level family-wise error (FWE) correction (Eickhoff et al., 2016) to correct for multiple comparisons. The meta-analyses were performed with a cluster-level FWE correction set at *p* < 0.05 (Müller et al., 2018), a threshold for forming clusters at *p* < 0.001 and 5,000 permutations. Additional exploratory meta-analyses were conducted for datasets that did not respect criteria of number of experiments (Müller et al., 2018): “*EB Spatial Navigation*” and *“SC Spatial Navigation.”* For these analyses, the threshold for forming clusters was set at *p* < 0.01. Thus, it is important to state that results from these exploratory analyses with less conservatory thresholds constitute preliminary data that should be more thoroughly investigated in future studies. As for the contrasts meta-analyses, they were performed with a significance level set at *p* < 0.01 and 1,000 permutations (Eickhoff et al., 2011). Finally, the conjunction meta-analyses, detecting the overlap between two ALE maps, were performed with a significance level set at *p* < 0.01 and 1,000 permutations (Eickhoff et al., 2011). The obtained results were overlaid on the Colin_27_T1_seg_MNI template (see text footnote 2) using Mango.^2^ Reported coordinates are in MNI space.

## Results

Results obtained from the performed meta-analyses report the neural correlates of spatial navigation and spatial processing in 31 neuroimaging studies, spanning more than 20 years of research. As mentioned above, as spatial navigation tasks usually integrate spatial and decision-making tasks, we separated *spatial processing* and *spatial navigation* analyses and presented them individually. However, due to the limited number of studies investigating spatial navigation, we performed an additional “*Spatial Processing* + *Spatial Navigation”* analysis, combining spatial processing and spatial navigation studies, which revealed extra clusters when compared to the “*Spatial Processing”* meta-analysis.

### Spatial processing analysis

**FIGURE 2:**
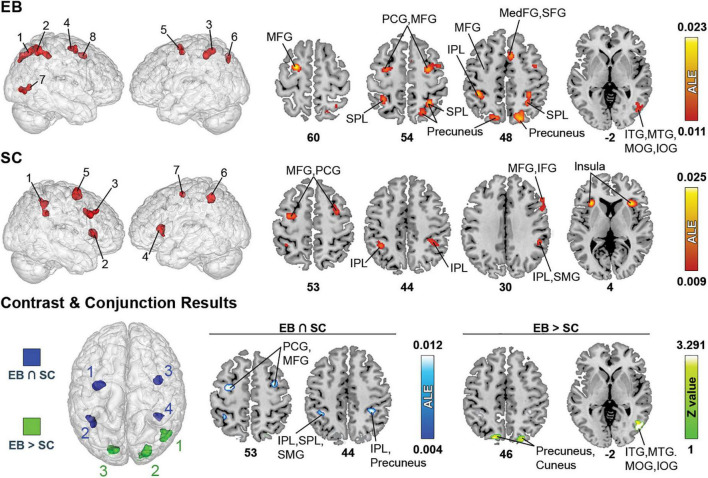
Brain areas activated by spatial processing in EB and SC and results of contrast and conjunction analyses. **(Left)** 3D renders of the brain and activation clusters (see Table 4 for reference). **(Right)** Axial cuts of the brain with identified clusters of activation. The number below the slices refer to the z coordinate in MNI space. ALE (for single and conjunction meta-analyses) and Z values (for contrast meta-analyses) are displayed to the right. The ALE values, calculated for each voxel, represent the probability of activation across included studies (Turkeltaub et al., 2012). For the contrast analysis, Z-values show the significance of ALE subtractions between two groups. More details regarding the ALE and Z values are presented in Table 3. IOG, inferior occipital gyrus; IPL, inferior parietal lobule; ITG, inferior temporal gyrus; MedFG, medial frontal gyrus; MFG, middle frontal gyrus; MTG, medial temporal gyrus; PCG, precentral gyrus; SFG, superior frontal gyrus; SMG, supramarginal gyrus; SPL, superior parietal lobule.

The spatial processing analysis included many spatial tasks such as sound localization, motion processing, auditory distance judgments, and auditory spatial working memory. Detailed information of all clusters is shown in Table 3 and Figure 2.

**TABLE 3:** Brain clusters obtained from the activation likelihood estimation (ALE) meta-analysis for spatial processing and contributing studies.

# Cluster	Brain regions (% of cluster volume)	Brodmann areas (% of cluster volume)	Cluster center (MNI x,y,z)	Cluster size (mm^3^)	Max ALE or Z value	Contributing studies
**EB**						
1	Precuneus (69%), Cuneus (28%), SPL (3%)	7 (62%), 19 (36%), 18 (2%)	(20,-73,47)	3,608	0.0203	Weeks et al., 2000; Gougoux et al., 2005; Ptito and Kupers, 2005; Stilla et al., 2008; Voss et al., 2008; Renier et al., 2010; Collignon et al., 2011; Voss et al., 2011; Anurova et al., 2015; Tao et al., 2015
2	SPL (52%), Precuneus (42%), IPL (4%)	7 (93%), 40 (7%)	(33, –47,48)	2,960	0.0197	Weeks et al., 2000; Gougoux et al., 2005; Stilla et al., 2008; Voss et al., 2008; Matteau et al., 2010; Renier et al., 2010; Collignon et al., 2011; Voss et al., 2011; Bonino et al., 2015; Dormal et al., 2016
3	IPL (54%), SPL (24%), SMG (11%), Precuneus (11%)	40 (65%), 7 (35%)	(–35, –44,49)	2,096	0.0213	Weeks et al., 2000; Matteau et al., 2010; Renier et al., 2010; Collignon et al., 2011; Park et al., 2011; Anurova et al., 2015; Tao et al., 2015; Dormal et al., 2016
4	PCG (58%), MFG (42%)	6 (100%)	(34, –4,53)	1,920	0.0185	Gougoux et al., 2005; Ricciardi et al., 2007; Stilla et al., 2008; Voss et al., 2008; Renier et al., 2010; Collignon et al., 2011; Voss et al., 2011; Striem-Amit et al., 2012; Anurova et al., 2015; Tao et al., 2015
5	MFG (71%), PCG (22%), Sub-gyral (7%)	6 (98%), 4 (1%)	(–25, –6,56)	1,808	0.0226	Stilla et al., 2008; Matteau et al., 2010; Renier et al., 2010; Collignon et al., 2011; Anurova et al., 2015; Tao et al., 2015
6	Precuneus (78%), Cuneus (22%)	7 (86%), 19 (14%)	(–15, –74,46)	1,576	0.0163	Weeks et al., 2000; Ptito and Kupers, 2005; Ricciardi et al., 2007; Stilla et al., 2008; Renier et al., 2010; Chan et al., 2012; Tao et al., 2015
7	ITG (30%), IOG (25%), MTG (25%), MOG (21%),	19 (45%), 37 (40%)	(49, –68, –2)	1,424	0.0146	Gougoux et al., 2005; Bonino et al., 2008; Voss et al., 2008; Ptito et al., 2009; Renier et al., 2010; Voss et al., 2011; Anurova et al., 2015; Dormal et al., 2016
8	MedFG (51%), Cingulate Gyrus (25%), SFG (24%),	6 (54%), 32 (21%), 24 (19%), 8 (5%)	(7,12,48)	4,136	0.0184	Ricciardi et al., 2007; Stilla et al., 2008; Matteau et al., 2010; Renier et al., 2010; Anurova et al., 2015; Tao et al., 2015
**SC**						
1	IPL (88%), SMG (9%), Sub-Gyral (3%)	40 (100%)	(44, –42,38)	2,208	0.0156	Weeks et al., 2000; Ptito et al., 2009; Matteau et al., 2010; Renier et al., 2010; Collignon et al., 2011; Chan et al., 2012; Anurova et al., 2015; Dormal et al., 2016
2	Insula (67%), Claustrum (24%), IFG (9%)	13 (74%), 47 (1%), 45 (1%)	(36,22,4)	2,080	0.0248	Ptito et al., 2005; Gougoux et al., 2005; Voss et al., 2008; Ptito et al., 2009; Bedny et al., 2010; Matteau et al., 2010; Renier et al., 2010; Voss et al., 2011; Anurova et al., 2015
2	MFG (53%), PCG (25%), Sub-Gyral (18%), SFG (5%)	6 (100%)	(32,1,58)	2,064	0.0221	Gougoux et al., 2005; Ricciardi et al., 2007; Voss et al., 2008; Ptito et al., 2009; Renier et al., 2010; Collignon et al., 2011; Park et al., 2011; Voss et al., 2011; Dormal et al., 2016
4	Insula (64%), Claustrum (36%)	13 (64%)	(–32,21,5)	1,600	0.0238	Gougoux et al., 2005; Ptito et al., 2005; Voss et al., 2008; Bedny et al., 2010; Matteau et al., 2010; Renier et al., 2010; Voss et al., 2011; Anurova et al., 2015
5	IFG (57%), MFG (34%), PCG (9%)	9 (90%), 6 (9%), 8 (1%)	(57,15,30)	1,520	0.0148	Gougoux et al., 2005; Ricciardi et al., 2007; Voss et al., 2008; Bedny et al., 2010; Voss et al., 2011; Chan et al., 2012; Bonino et al., 2015; Dormal et al., 2016
6	IPL (83%), SMG (15%), SPL (2%)	40 (98%), 7 (2%)	(–37, –47,46)	1,368	0.0159	Gougoux et al., 2005; Voss et al., 2008; Renier et al., 2010; Park et al., 2011; Voss et al., 2011; Dormal et al., 2016
7	PCG (62%), MFG (38%)	6 (88%), 4 (12%)	(–30, –8,53)	1,072	0.0168	Matteau et al., 2010; Collignon et al., 2011; Anurova et al., 2015; Dormal et al., 2016
**EB > SC**						
1	ITG (30%), IOG (25%), MTG (25%), MOG (20%)	19 (45%), 37 (40%)	(50, –69, –3)	1,256	3.29 Z	Gougoux et al., 2005; Bonino et al., 2008; Voss et al., 2008; Ptito et al., 2009; Renier et al., 2010; Voss et al., 2011; Anurova et al., 2015
2	Precuneus (58%), Cuneus (42%)	19 (61%), 7 (32%), 18 (7%)	(21, –80,44)	1,024	3.29 Z	Ptito et al., 2005); Renier et al., 2010
3	Precuneus (97%), Cuneus (3%)	7 (89%), 19 (11%)	(–14, –76,46)	648	3.29 Z	Ptito et al., 2005; Tao et al., 2015
**SC > EB**						
**NA**						
**EB ∩ SC**						
1	MFG (53%), PCG (47%)	6 (97%), 4 (3%)	(–29, –8,53)	736	0.0159	Matteau et al., 2010; Renier et al., 2010; Collignon et al., 2011; Dormal et al., 2016
2	IPL (83%), SMG (15%), SPL (2%)	40 (98%), 7 (2%)	(–37, –45,47)	688	0.0142	Weeks et al., 2000; Renier et al., 2010; Collignon et al., 2011; Dormal et al., 2016
3	PCG (58%), MFG (42%)	6 (100%)	(33, –3,54)	488	0.014	Ptito et al., 2009; Renier et al., 2010; Collignon et al., 2011
4	Precuneus	7	(35, –41,44)	456	0.013	Matteau et al., 2010; Collignon et al., 2011; Dormal et al., 2016

MFG, middle frontal gyrus; MedFG, medial frontal gyrus; MOG, middle occipital gyrus; MTG, middle temporal gyrus; IFG, inferior frontal gyrus; IOG, inferior occipital gyrus; IPL, inferior parietal lobule; ITG, inferior temporal gyrus; SFG, superior frontal gyrus; SMG, supramarginal gyrus; SPL, superior parietal lobule.

#### Single meta-analyses

The ALE analysis revealed eight significant clusters in EB that were located in parietal, (pre)frontal, temporal, and occipital gyri. Parietal clusters included the precuneus, superior parietal lobule (SPL), inferior parietal lobule (IPL), supramarginal gyrus (SMG). Clusters in the (pre)frontal lobe included the precentral gyrus (PCG), middle frontal gyrus (MFG), right medial frontal gyrus (MedFG), superior frontal gyrus (SFG) and anterior cingulate cortex (ACC). Temporal clusters included the middle and inferior temporal gyri (MTG and ITG). Occipital clusters included the cuneus, and right middle and inferior occipital gyri (MOG and IOG). The ALE analysis revealed seven clusters in SC, located in parietal and (pre)frontal lobes, as well as in sub-lobar structures. Parietal clusters included the IPL, SMG, and left SPL. Clusters in the (pre)frontal lobe included the PCG, MFG, right inferior frontal gyrus (IFG), and right SFG. Sub-lobar clusters included the insula and claustrum. As stated previously, given the paucity of studies strictly dealing with LB populations, no datasets were possible in this category.

#### Contrast and conjunction meta-analyses

Contrast meta-analyses revealed that EB recruited a larger network than SC. These included the precuneus, cuneus, MOG, IOG, MTG, and ITG. The conjunction analysis revealed shared activations in EB and SC in precuneus, IPL, SMG, MFG and PCG.

### Exploratory spatial navigation analysis

**FIGURE 3:**
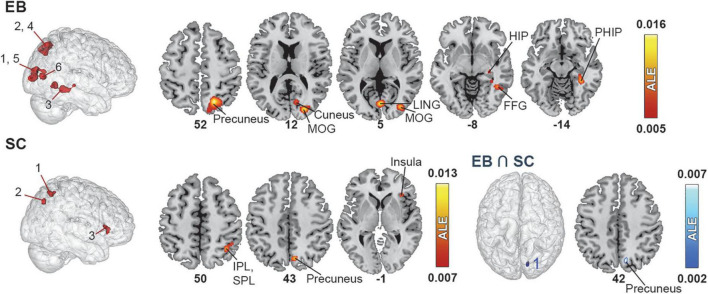
Brain areas activated by spatial navigation in EB and SC and results of conjunction analysis. **(Left)** 3D renders of the brain and activation clusters (see Table 4 for reference). **(Right)** Axial cuts of the brain with identified clusters of activation. The number below the slices refer to the z coordinate in MNI space. The ALE values, calculated for each voxel, represent the probability of activation across included studies (Turkeltaub et al., 2012) and are disclosed to the right. More details regarding the ALE values are presented in Table 4. FFG, fusiform gyrus; HIP, hippocampus; IPL, inferior parietal lobule; LING, lingual gyrus; MOG, middle occipital gyrus; PHIP, parahippocampus.

The spatial navigation exploratory analysis included tasks such as route recognition, maze learning, and discrimination of spatial layouts and landmark objects/features through various means such as SSDs, echolocation, and tactile exploration. Detailed information about all clusters can be found in Table 4 and Figure 3.

**TABLE 4:** Brain clusters obtained from the activation likelihood estimation (ALE) meta-analysis for spatial navigation and contributing studies.

# Cluster	Brain regions (% of cluster volume)	Brodmann areas (% of cluster volume)	Cluster center (MNI x,y,z)	Cluster size (mm^3^)	Max ALE value	Contributing studies
**EB**						
1*	Cuneus (42%), Lingual Gyrus (36%), MOG (19%), IOG (2%)	18 (52%), 17 (30%), 23 (7%), 30 (4%)	(19, –82,8)	5,992	0.0152	Kupers et al., 2010; Struiksma et al., 2011; Gagnon et al., 2012; Halko et al., 2014; Milne et al., 2015; Dodsworth, 2019
2*	Precuneus (79%), SPL (12%), Cuneus (9%)	7 (79%), 19 (21%)	(18, –70,49)	4,728	0.0158	Kupers et al., 2010; Wolbers et al., 2011; Gagnon et al., 2012; Halko et al., 2014; Fiehler et al., 2015
3*	Parahippocampal Gyrus (55%), Fusiform Gyrus (45%)	37 (56%), 36 (33%), 20 (6%), 19 (5%)	(38, –43, –11)	2,600	0.0125	Kupers et al., 2010; Gagnon et al., 2012; He et al., 2013
4	Precuneus (84%), SPL (15%), Cuneus (2%)	7 (97%), 19 (3%)	(18, –68,50)	1,880	0.0158	Kupers et al., 2010; Wolbers et al., 2011; Gagnon et al., 2012; Halko et al., 2014; Fiehler et al., 2015
5	Cuneus (52%), MOG (45%), Lingual Gyrus (3%)	17 (55%), 18 (45%)	(29, –85,9)	1,600	0.0151	Kupers et al., 2010; Gagnon et al., 2012; Dodsworth, 2019
6	Lingual Gyrus (93%), Cuneus (7%)	18 (96%)	(1, –78,5)	928	0.0152	Kupers et al., 2010; Struiksma et al., 2011; Gagnon et al., 2012
**SC**						
1	IPL (58%), SPL (42%)	7 (55%), 40 (45%)	(38, –55,50)	808	0.0108	Kupers et al., 2010; Struiksma et al., 2011; Fiehler et al., 2015
2	Precuneus (87%), Cuneus (13%)	7 (100%)	(8, –71,43)	752	0.012	Kupers et al., 2010; Gagnon et al., 2012
3	Insula (58%), IFG (21%), Claustrum (21%)	13 (52%), 47 (12%), 45 (9%)	(35,25, –1)	744	0.0094	Kupers et al., 2010; Struiksma et al., 2011; Fiehler et al., 2015; Dodsworth, 2019
**EB > SC**						
NA						
**SC > EB**						
NA						
**EB ∩ SC**						
1	Precuneus (100%)	7 (100%)	(11, –71,42)	144	0.0091	Gagnon et al., 2012

*Thresholded at *P* < 0.01. IFG, inferior frontal gyrus; IOG, inferior occipital gyrus; IPL, inferior parietal lobule; MOG, middle occipital gyrus; SPL, superior parietal lobule.

#### Single meta-analyses

The ALE analysis revealed six clusters in EB located in the parietal, occipital, and temporal cortices in the right hemisphere. Parietal clusters included the precuneus and SPL. Occipital clusters included the cuneus, MOG, IOG and lingual gyrus. The temporal cluster included the fusiform gyrus and parahippocampal gyrus (PHIP) and extended to a portion of the hippocampus (HIP). The ALE analysis revealed three significant clusters of activation in the right hemisphere in SC. The first cluster included the SPL and IPL; the second, the precuneus and cuneus; and the third, the insula, claustrum and IFG. No LB datasets were possible in this category.

#### Contrast and conjunction meta-analyses

Given the small number of studies in the *spatial navigation* datasets, the contrast analysis revealed no significant clusters that were more activated in either EB or SC. The conjunction meta-analysis identified one small (144 mm^3^) cluster in the precuneus that was commonly activated by EB and SC.

### Spatial processing + spatial navigation analyses

**FIGURE 4:**
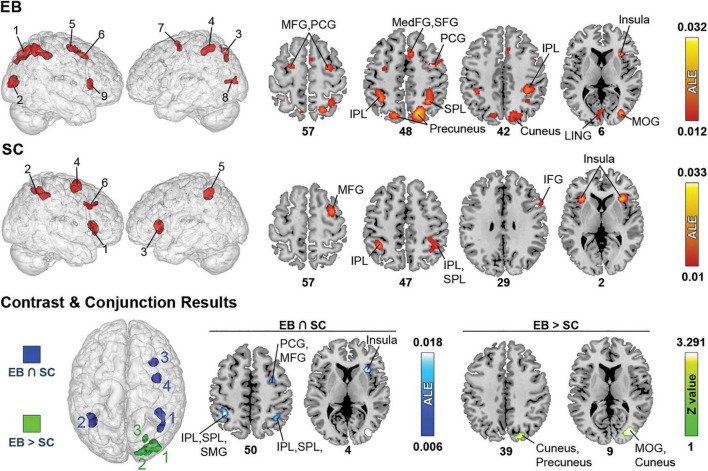
Brain areas activated in spatial processing + spatial navigation in EB, LB, and SC and results of contrast and conjunction analyses. **(Left)** 3D renders of the brain and activation clusters (see Table 4 for reference). **(Right)** Axial cuts of the brain with identified clusters of activation. The number below the slices refer to the z coordinate in MNI space. ALE (for single and conjunction meta-analyses) and Z values (for contrast meta-analyses) are displayed to the right. The ALE values, calculated for each voxel, represent the probability of activation across included studies (Turkeltaub et al., 2012). For the contrast analysis, Z-values are used to show the significance of ALE subtractions between two groups. More details regarding the ALE and Z values are presented in Table 5. IOG, inferior occipital gyrus; IPL, inferior parietal lobule; ITG, inferior temporal gyrus; LING, lingual gyrus; MedFG, medial frontal gyrus; MFG, middle frontal gyrus; MOG, middle occipital gyrus; MTG, medial temporal gyrus; PCG, precentral gyrus; SFG, superior frontal gyrus; SPL, superior parietal lobule.

Here, we performed ALE analyses on *spatial processing* and *spatial navigation* tasks combined. Detailed information about all significant clusters can be found in Table 5 and Figure 4.

**TABLE 5:** Brain clusters obtained from the activation likelihood estimation (ALE) meta-analysis for spatial processing + spatial navigation and contributing studies.

# Cluster	Brain regions (% of cluster volume)	Brodmann areas (% of cluster volume)	Cluster center (MNI x,y,z)	Cluster size (mm^3^)	Max ALE or Z value	Contributing studies
**EB**						
1	Precuneus (58%), Cuneus (20%), SPL (19%), IPL (3%)	7 (72%), 19 (22%), 40 (3%), 18 (2%), 31 (1%)	(25, –62,48)	11,248	0.0319	Weeks et al., 2000; Gougoux et al., 2005; Ptito et al., 2005; Stilla et al., 2008; Voss et al., 2008; Kupers et al., 2010; Matteau et al., 2010; Renier et al., 2010; Collignon et al., 2011; Park et al., 2011; Struiksma et al., 2011; Voss et al., 2011; Wolbers et al., 2011; Gagnon et al., 2012; Halko et al., 2014; Anurova et al., 2015; Bonino et al., 2015; Fiehler et al., 2015; Tao et al., 2015; Dormal et al., 2016
2	MOG (73%), Cuneus (27%)	18 (55%), 17 (27%), 19 (19%)	(32, –84,9)	3,168	0.0255	Weeks et al., 2000; Stilla et al., 2008; Ptito et al., 2009; Kupers et al., 2010; Matteau et al., 2010; Gagnon et al., 2012; Anurova et al., 2015; Dodsworth, 2019
3	Precuneus (70%), Cuneus (31%)	7 (82%), 19 (15%), 18 (4%)	(–14, –74,47)	2,448	0.0213	Chan et al., 2012; Tao et al., 2015; Weeks et al., 2000; Stilla et al., 2008; Ptito et al., 2005; Ricciardi et al., 2007; Kupers et al., 2010; Gagnon et al., 2012; Wolbers et al., 2011
4	IPL (61%), SPL (25%), Precuneus (8%), SMG (6%)	40 (64%), 7 (36%)	(–35, –46,49)	2,400	0.0254	Weeks et al., 2000; Kupers et al., 2010; Matteau et al., 2010; Renier et al., 2010; Collignon et al., 2011; Park et al., 2011; Struiksma et al., 2011; Anurova et al., 2015; Tao et al., 2015; Dormal et al., 2016
5	PCG (59%), MFG (40%)	6 (100%)	(35, –3,52)	2,304	0.024	Gougoux et al., 2005; Bonino et al., 2008; Stilla et al., 2008; Voss et al., 2008; Renier et al., 2010; Collignon et al., 2011; Striem-Amit et al., 2012; Struiksma et al., 2011; Voss et al., 2011; Anurova et al., 2015; Fiehler et al., 2015; Tao et al., 2015; Dormal et al., 2016
6	MedFG (63%), Cingulate Gyrus (19%), SFG (19%)	6 (63%), 32 (24%), 24 (13%)	(7,12,49)	1,536	0.019	Ricciardi et al., 2007; Stilla et al., 2008; Kupers et al., 2010; Renier et al., 2010; Collignon et al., 2011; Park et al., 2011; Struiksma et al., 2011; Anurova et al., 2015; Tao et al., 2015
7	MFG (67%), PCG (26%), Sub-Gyral (7%)	6 (97%), 4 (3%)	(–26, –6,56)	1,528	0.0227	Stilla et al., 2008; Matteau et al., 2010; Renier et al., 2010; Collignon et al., 2011; Anurova et al., 2015; Tao et al., 2015
8	Lingual gyrus (72%), Cuneus (28%)	18 (46%), 17 (44%)	(–3, –86,7)	1,480	0.0166	Gougoux et al., 2005; Voss et al., 2008; Ptito et al., 2009; Kupers et al., 2010; Collignon et al., 2011; Struiksma et al., 2011; Voss et al., 2011; Gagnon et al., 2012; Anurova et al., 2015
9	Claustrum (59%), Insula (41%)	13 (41%)	(34,20,5)	1,304	0.0242	Gougoux et al., 2005; Voss et al., 2008; Kupers et al., 2010; Matteau et al., 2010; Renier et al., 2010; Park et al., 2011; Struiksma et al., 2011; Voss et al., 2011; Anurova et al., 2015
**SC**						
1	Insula (62%), Claustrum (22%), IFG (15%), Extra-Nuclear (2%)	13 (60%), 47 (7%), 45 (4%)	(36,23,2)	3,000	0.033	Gougoux et al., 2005; Ptito et al., 2005; Voss et al., 2008; Ptito et al., 2009; Bedny et al., 2010; Kupers et al., 2010; Matteau et al., 2010; Renier et al., 2010; Struiksma et al., 2011; Voss et al., 2011; Anurova et al., 2015; Fiehler et al., 2015; Dodsworth, 2019
2	IPL (79%), SPL (18%), SMG (2%)	40 (78%), 7 (22%)	(40, –47,47)	2,984	0.0174	Gougoux et al., 2005; Ptito et al., 2005; Voss et al., 2008; Kupers et al., 2010; Matteau et al., 2010; Struiksma et al., 2011; Voss et al., 2011; Chan et al., 2012; Anurova et al., 2015; Fiehler et al., 2015; Dormal et al., 2016
3	Insula (65%), Claustrum (29%), extra-nuclear (3%), IFG (2%)	13 (60%), 47 (5%)	(–32,21,3)	2,400	0.0279	Ptito and Kupers, 2005; Gougoux et al., 2005; Voss et al., 2008; Matteau et al., 2010; Bedny et al., 2010; Kupers et al., 2010; Renier et al., 2010; Voss et al., 2011; Struiksma et al., 2011; Anurova et al., 2015; Dodsworth, 2019
4	MFG (55%), PCG (25%), Sub-Gyral (16%), SFG (4%)	6 (100%)	(32,0,57)	2,392	0.0233	Gougoux et al., 2005; Ricciardi et al., 2007; Voss et al., 2008; Ptito et al., 2009; Renier et al., 2010; Collignon et al., 2011; Park et al., 2011; Struiksma et al., 2011; Voss et al., 2011; Dormal et al., 2016
5	IPL (78%), SMG (18%), SPL (3%), Sub-Gyral (1%)	40 (98%), 7 (2%)	(–37, –47,46)	2,288	0.0232	Weeks et al., 2000; Gougoux et al., 2005; Voss et al., 2008; Renier et al., 2010; Kupers et al., 2010; Park et al., 2011; Struiksma et al., 2011; Voss et al., 2011; Fiehler et al., 2015; Dormal et al., 2016
6	IFG (64%), MFG (31%), PCG (6%),	9 (94%), 6 (6%)	(57,17,29)	1,000	0.0148	Ricciardi et al., 2007; Bedny et al., 2010; Chan et al., 2012; Bonino et al., 2015; Dormal et al., 2016
**EB > SC**						
1	MOG (83%), Cuneus (18%)	18 (62%), 19 (21%), 17 (18%)	(33, –84,9)	3,008	3.29 Z	Weeks et al., 2000; Stilla et al., 2008; Ptito et al., 2009; Kupers et al., 2010; Gagnon et al., 2012; Anurova et al., 2015; Dodsworth, 2019
2	Cuneus (67%), Precuneus (33%)	19 (53%), 7 (29%), 18 (12%), 31 (5%)	(21, –82,39)	1,416	3.29 Z	Renier et al., 2010; Collignon et al., 2011; Wolbers et al., 2011; Dormal et al., 2016
3	Precuneus	7	(20, –66,52)	224	2.65 Z	Stilla et al., 2008; Renier et al., 2010; Gagnon et al., 2012; Halko et al., 2014
**SC > EB**						
NA						
**EB ∩ SC**						
1	SPL (75%), IPL (25%)	7 (83%), 40 (17%)	(36, –45,46)	1,504	0.0171	Kupers et al., 2010; Matteau et al., 2010; Collignon et al., 2011; Struiksma et al., 2011; Fiehler et al., 2015; Dormal et al., 2016
2	IPL (85%), SMG (9%), SPL (6%)	40 (96%), 7 (4%)	(–36, –46,48)	1,208	0.021	Weeks et al., 2000; Gougoux et al., 2005; Voss et al., 2008; Kupers et al., 2010; Renier et al., 2010; Collignon et al., 2011; Park et al., 2011; Struiksma et al., 2011; Voss et al., 2011; Anurova et al., 2015; Fiehler et al., 2015; Dormal et al., 2016
3	Claustrum (56%), Insula (44%)	13 (44%)	(34,20,4)	1,072	0.0242	Ptito et al., 2009; Bedny et al., 2010; Kupers et al., 2010; Matteau et al., 2010; Park et al., 2011; Renier et al., 2010; Struiksma et al., 2011; Anurova et al., 2015
4	PCG (52%), MFG (48%)	6 (100%)	(31, –3,54)	728	0.0181	Stilla et al., 2008; Ptito et al., 2009; Renier et al., 2010; Collignon et al., 2011; Struiksma et al., 2011; Bonino et al., 2015

MFG, middle frontal gyrus; MedFG, medial frontal gyrus; MOG, middle occipital gyrus; IFG, inferior frontal gyrus; IPL, inferior parietal lobule; PCG, precentral gyrus; SFG, superior frontal gyrus; SMG, supramarginal gyrus; SPL, superior parietal lobule.

#### Single meta-analyses

The ALE analysis revealed nine significant clusters in EB, located in parietal, (pre)frontral, and occipital lobes, as well as sub-lobar structures. In the parietal lobe, clusters included the precuneus, SPL, and IPL. Clusters in the (pre)frontal lobe covered the MFG, PCG, right MedFG, right SFG, and right ACC. Clusters in the occipital lobe included the cuneus, lingual gyrus, and right MOG. Clusters in sub-lobar structures included the insula and claustrum. For SC, six clusters were found in the parietal and (pre)frontal lobes, and in sub-lobar structures. In the parietal lobe, these included the IPL, SPL and SMG. Clusters in the (pre)frontal lobe included the IFG and right MFG, PCG, and SFG. Sub-lobar clusters included the insula and claustrum.

#### Contrast and conjunction meta-analyses

Only one contrast meta-analysis (EB > SC) produced significant results and revealed that EB recruited a larger network of brain structures than their SC counterparts. This network included the precuneus, cuneus, MOG and lingual gyrus. Conjunction meta-analyses revealed shared clusters between EB and SC in SPL, IPL, insula, claustrum, MFG and PCG. Clusters common to EB and LB were located in the left MFG and PCG. There were no shared clusters common for LB and SC.

### Qualitative review of neural activity in late blind

There was an insufficient number of studies to conduct a meta-analysis in LB. We therefore performed a qualitative analysis of the literature. The results show that LB had similar activity in parietal and (pre)frontal cortices (Stilla et al., 2008; Tao et al., 2015, 2017); however, there was a lack of consensus on the presence and magnitude of occipital activation during spatial tasks. On the one hand, occipital activation in EB and LB were found during tactile drawing (Likova, 2012), tactile microspatial discrimination (Stilla et al., 2008), and auditory localization (Voss et al., 2006, 2011; Collignon et al., 2013). Moreover, this activation often correlated with performance (Collignon et al., 2013) or level of expertise in the task (Thaler et al., 2011; Norman and Thaler, 2019). On the other hand, such visual occipital activity was superior in EB (Collignon et al., 2013; Tao et al., 2015, 2017; Jiang et al., 2016) or was only present in EB (Voss et al., 2008). In terms of spatial patterns of observed activations, LB often showed activations of the lateral occipital cortex, MOG, lingual gyrus, and cuneus (Voss et al., 2006, 2011; Stilla et al., 2008; Tao et al., 2017). LB also showed greater reliance on the precuneus and other parietal and (pre)frontal areas (Tao et al., 2015, 2017), in line with results obtained in SC. In conclusion, consistent with the idea of cross-modal plasticity being experience-driven (Ptito et al., 2021a), LB often show activations that are intermediary between EB and SC patterns.

## Discussion

In the present study, we used ALE meta-analyses to identify brain regions commonly activated in neuroimaging studies of non-visual spatial processing and navigation in blind and sighted individuals. Our analyses identified supramodal brain areas that are activated by spatial tasks in both EB and SC. In addition, we identified occipital brain areas that are cross-modally recruited by blind individuals. Due to the limited literature on LB (nine studies, six contrasts), we could not identify the neural correlates of spatial processing and spatial navigation in this population. The next sections will thus strongly focus on findings in EB and SC populations.

### Neural correlates of non-visual spatial processing

The ALE analysis revealed that non-visual spatial information processing via tactile and auditory modalities activates a neural network that comprises dorsal frontal and parietal regions in both hemispheres. This core network was shown to be recruited in both SC and EB participants and includes the SPL, IPL, MFG, MedFG, and pre-central gyrus. A shared activation of the right insula in EB and SC was also identified when navigation tasks were included in the meta-analysis. Furthermore, the ALE analysis revealed that EB individuals also activated the pre-SMA and cross-modally recruited occipital areas during spatial tasks. These activations are similar to those reported in previous meta-analyses in blind (Zhang et al., 2019) and sighted individuals (Parlatini et al., 2017; Cona and Scarpazza, 2019). Indeed, Zhang et al. (2019) also reported that blind participants consistently activated precuneus, SPL, IPL, MFG, pre-central gyrus, insula, cuneus and other occipital areas along with extra parahippocampal gyrus activations. Taken together, these meta-analyses indicate that both blind and sighted participants recruit two interlinked frontoparietal networks for performing spatial tasks, a dorsal frontoparietal network, also known as the dorsal attention network (DAN), and a ventral frontoparietal network.

The DAN includes the SPL, intraparietal sulcus (IPS), dorsal premotor areas and frontal eye fields (FEF), an area located around the intersection of the precentral gyrus and MFG (Corbetta et al., 2008; Power et al., 2011; Vernet et al., 2014). The DAN mediates both spatial attention and spatial working memory and allows for the selection of sensory stimuli that are not only salient, but also relevant to the individual’s goals (top-down attention) in order to plan contextually appropriate responses (Serences and Yantis, 2007; Corbetta et al., 2008; Cona and Scarpazza, 2019). DAN regions are organized in topographic maps corresponding to areas of the visual field (Hagler and Sereno, 2006; Jerde and Curtis, 2013), most specifically to a range of polar angles and eccentricities (Mackey et al., 2017). It is therefore theorized that the DAN serves to implement internal spatial representations (i.e., priority maps of space) and to allocate top-down attention toward them (Cona and Scarpazza, 2019). Our meta-analysis suggests that these internal spatial representations in DAN regions are not necessarily organized according to the visual field but are in fact *amodal*, and independent from visual experience. The most commonly activated DAN regions in the ALE analysis were the SPL and dorsal premotor areas including the FEF (Paus, 1996; Luna et al., 1998; Ioannides et al., 2004; Garg et al., 2007; Vernet et al., 2014). The SPL is classically associated with visual guidance of actions (Karnath and Perenin, 2005; Prado et al., 2005) and visuospatial attention (Silver et al., 2005; Saygin and Sereno, 2008; Vandenberghe et al., 2012; Wu et al., 2016), and codes space in an egocentric reference frame, including the known space outside the visual field such as the rear space (Schindler and Bartels, 2013). The FEF is strongly involved in the control of different types of eye movements (mostly saccadic eye movements, but also fixation, pursuit, and vergence), the orientation of attention, visual awareness and perceptual modulation (Moore and Fallah, 2001; Squire et al., 2013; Vernet et al., 2014). However, the SPL and FEF are also recruited in non-visual spatial tasks such as auditory distance judgments (Collignon et al., 2011), localization of sounds on the horizontal axis (Weeks et al., 2000; Gougoux et al., 2005; Renier et al., 2010; Anurova et al., 2015), covert allocation of auditory spatial attention (Garg et al., 2007), auditory motion processing (Dormal et al., 2016), auditory spatial working memory (Park et al., 2011) and tactile microspatial discrimination Stilla et al. (2008) in both blind and sighted individuals. It is therefore proposed that these regions most likely code peripersonal space based on the available or contextually relevant sensory information. Whereas in sighted persons this is mostly based on visual information, blind individuals primarily rely on haptic and auditory cues.

The ventral frontoparietal network includes the temporo-parietal junction (encompassing IPL and superior temporal sulcus), parts of the middle and inferior frontal gyri, the frontal operculum, and the insula. This network is associated with bottom-up attention; it mediates the detection of unattended salient and behaviorally relevant stimuli, and consequently guides actions (Seeley et al., 2007; Corbetta et al., 2008; Cona and Scarpazza, 2019). The ALE revealed activations of the IPL and insula. The IPL is a multimodal brain region that is mostly involved in the detection of salient novel stimuli, in sustaining attention (Singh-Curry and Husain, 2009), and in motor imagery (Kraeutner et al., 2019). Studies in the macaque monkey demonstrated that the IPL is involved in the transformation of information from all sensory modalities into motor behaviors (Borra and Luppino, 2017; Niu et al., 2021). This is corroborated by studies in humans that have identified numerous connections between IPL and frontal, occipital and temporal regions (Caspers et al., 2011; Sun et al., 2022). The insula was the other node of the ventral frontoparietal network that was identified by the ALE analysis. The insula is involved in prioritizing stimuli and spatial maps in the DAN. This is based on both bottom-up inputs and top-down internal information from higher association cortices including, but not limited to, the individual’s goals and previous sensory input (Serences and Yantis, 2007; Myers et al., 2017).

Similarly to findings from Zhang et al. (2019), we also found that EB recruited a larger network than SC including the precuneus, cuneus, lingual gyrus, and MTG. Moreover, we found additional activations in the pre-SMA, MOG, IOG, and ITG. The pre-SMA activation might be explained by its extensive role in the sequential integration of spatial information to form higher order representations (Cona and Semenza, 2017; Cona and Scarpazza, 2019). Furthermore, there is now ample evidence that the cuneus, lingual gyrus, MOG, IOG, MTG and ITG are cross-modally recruited by other senses, and thus maintain their spatial function in EB individuals (Ricciardi et al., 2007; Ptito et al., 2009; Matteau et al., 2010; Renier et al., 2010; Collignon et al., 2011; Dormal et al., 2016; Huber et al., 2019); they might even serve a multisensory role in normal sighted subjects (Palejwala et al., 2021). This could explain why in the absence of vision, the dorsal visual stream appears to be preserved both structurally (Reislev et al., 2016) and functionally (Fiehler et al., 2009; Zhang et al., 2019).

There is some evidence that the cross-modal recruitment of occipital areas in EB is topographically organized, forming new “retinotopic-like” cortical maps. These include new cortical representations of the fingers in the occipital cortex of Braille reading experts (Ptito et al., 2008a), and of the tongue in blind individuals trained with the tongue display unit (Kupers et al., 2006). Spatiotopic representations of auditory information in individuals trained in echolocation (Thaler et al., 2011; Arnott et al., 2013; Norman and Thaler, 2019; Van der Heijden et al., 2020) or trained with auditory SSDs (Hofstetter et al., 2021) have also been reported. It therefore seems that in the blind, visual areas in the occipital cortex maintain their function and form lower-level cortical maps of the perceived sensory information, similarly to frontoparietal networks. These new cortical maps are mostly egocentric and comparable to the retinotopic maps found in sighted subjects (Linton, 2021). It is theorized that this cross-modal recruitment in EB might arise from: (1) strengthened cortico-cortical connections between the occipital cortex with other sensory cortices and parietal associative areas (Wittenberg et al., 2004; Ptito et al., 2005; Kupers et al., 2006); or (2) new connections between the sensory nuclei of the thalamus enabling non-visual sensory information to arrive directly to the occipital cortex via the optic radiations (Kupers and Ptito, 2014; Müller et al., 2019).

### Neural correlates of non-visual spatial navigation

While previous meta-analyses (Ricciardi et al., 2014; Zhang et al., 2019) investigated spatial (navigation) tasks, they did not allow to distinguish between spatial processing and more complex spatial navigation tasks. As navigation integrates many tasks such as locomotion, echolocation, attention to stimuli, mental rotation, decision making, as well as working memory and long-term memory, this distinction is important for the present research question. Therefore, we also assessed the neural correlates of non-visual spatial navigation in EB individuals in one exploratory ALE analysis using a dataset of 11 contrasts. Clusters for spatial navigation included the precuneus, SPL, hippocampus, parahippocampal gyrus, fusiform gyrus, lingual gyrus and cuneus, all in the right hemisphere. Comparing with results from “*spatial processing* + *spatial navigation”* and “*spatial processing*,” an additional significant cluster was found in the right insula and claustrum. These results are in line with those of previous meta-analyses investigating the neural correlates of navigation in SC (Epstein et al., 2017; Cona and Scarpazza, 2019; Qiu et al., 2019; Li et al., 2021), giving further credit to the idea that SC and EB share the same neural networks, despite differences in sensory modalities and navigational strategies. Spatial navigation in SC has been related to large cortical and subcortical networks, including the medial temporal lobe (comprised of HIP and entorhinal, perirhinal and parahippocampal cortices), posterior parietal cortex, insula/claustrum, prefrontal cortex, and a “scene perception” network comprised of the parahippocampal place area (PPA), the retrosplenial complex (RSC), and occipital place area (OPA) (Epstein et al., 2017; Epstein and Baker, 2019; Qiu et al., 2019; Li et al., 2021).

The activations in EB of the precuneus and SPL during virtual navigation (Halko et al., 2014), route and path recognition (Kupers et al., 2010; Fiehler et al., 2015), and tactile maze learning (Gagnon et al., 2012) are consistent with results in SC in spatial tasks (Cona and Scarpazza, 2019). These areas are mostly involved in egocentric navigation, more specifically in coding the environment, objects, and landmarks around the individual, or along routes, to plan and execute movements in relation to them (Farrell, 1996; Farrell and Robertson, 2000; Milner and Goodale, 2006). Other studies reported that these areas are involved in mental navigation (Ghaem et al., 1997), imagining places or scenes (Bisiach et al., 1993; Burgess et al., 2001) and help sustaining navigation in virtual mazes (Ohnishi et al., 2006). Furthermore, route recognition (Kupers et al., 2010) and maze learning (Gagnon et al., 2012) was associated with activations of primary and secondary visual areas: the cuneus, MOG, and lingual gyrus, a region involved in the discrimination of direction and motion (Cornette et al., 1998) and spatial learning of an environment (Nemmi et al., 2013). The lingual gyrus is also linked to allocentric, as opposed to egocentric, navigational strategies (Li et al., 2021).

The next cluster included the hippocampus, parahippocampal gyrus and fusiform gyrus. This cluster was activated by route recognition (Kupers et al., 2010), maze learning (Gagnon et al., 2012), and the processing of tactile spatial layouts (Wolbers et al., 2011) and large non-manipulable objects (He et al., 2013). The hippocampus is extensively linked to spatial navigation as it is involved in the formation and use of cognitive maps (Hartley et al., 2003; Iaria et al., 2003, 2007; Marchette et al., 2011; Epstein et al., 2017). It contains spatial codes representing distance and time relationships in small and large environments (Hassabis et al., 2009; Morgan et al., 2011; Deuker et al., 2016) and its activity can predict navigational performance (Suthana et al., 2009). Volume of the right posterior hippocampus correlates with navigational performance, experience, and spatial learning (Woollett and Maguire, 2011; Hartley and Harlow, 2012; Schinazi et al., 2013). The portion of the cluster in the parahippocampal gyrus closely overlapped with the PPA (Park and Chun, 2009). The PPA is a region that shows stronger responses to (1) visual scenes containing spatial information relevant for navigation (Harel et al., 2013; Epstein et al., 2017; Epstein and Baker, 2019); and (2) location-related – or large non-manipulable – objects that may serve as environmental landmarks or be linked to decision points (Epstein et al., 1999; Janzen and Van Turennout, 2004; Schinazi and Epstein, 2010; Julian et al., 2017; Epstein and Baker, 2019). Consequently, the PPA is thought to have a role in encoding a representation of specific scenes and landmarks, enabling their recognition (Epstein, 2008).

The “scene perception” network, comprised of the PPA, RSC and OPA, subserves different roles in long-term spatial memory and navigation; damage to these areas causes wayfinding deficits (Aguirre and D’esposito, 1999). While the PPA is involved in the representation of spatial layouts of scenes, the RSC and OPA are involved in the representation of spatial relationships between the observer and the parts of a scene (Epstein and Baker, 2019). Activity in the RSC increases with the acquisition of spatial knowledge of an environment (Wolbers and Büchel, 2005) and codes for the allocentric heading direction (Spiers and Maguire, 2007; Epstein, 2008). It is believed that the RSC is more involved in providing allocentric representations, thus situating scenes within more extensive environments (Epstein and Higgins, 2007; Epstein et al., 2007; Epstein, 2008). However, there is less consensus about its role in spatial navigation. Whereas some authors suggest that the RSC may serve as a relay structure that converts allocentric spatial representations from the hippocampal complex to the egocentric representation in the posterior parietal cortex, others claim that it encodes and stores its own allocentric spatial representations (Epstein, 2008; Epstein and Baker, 2019). While specific clusters were not identified in the RSC, this region was activated by both visual and tactile spatial layouts in EB and SC (Wolbers et al., 2011). Taken together with PPA responses to scenes and spatially relevant information conveyed through visual, tactile and auditory stimuli (Kupers et al., 2010; Wolbers et al., 2011; Gagnon et al., 2012; Milne et al., 2015; Dodsworth, 2019) as well as through language (He et al., 2013), this result suggests that the spatial representations in this “scene perception” network may also be independent from visual experience and hence, amodal in nature.

Finally, activations of the anterior insula and claustrum, identified in the *spatial processing* + *spatial navigation* meta-analysis, are consistent with their known role in the processing goal-related sensory stimuli, in guiding behaviors, and in spatial learning (Hartley and Harlow, 2012). However, no other activations in (pre)frontal cortices were found even though they are associated with multiple wayfinding tasks such as switching navigational strategy, route planning, detours, and shortcuts (Cona and Scarpazza, 2019; Li et al., 2021). As it is the case for RSC, this null result may arise from the limited number of studies involving blind participants in such complex navigational tasks as only one study required participants to plan routes in a virtual environment (Halko et al., 2014).

### Amodal nature of spatial cognition and navigation

The present study adds to a growing body of research suggesting that the brain of EB and SC are similarly organized at the functional level (Proulx et al., 2014; Cecchetti et al., 2016; Arioli et al., 2021). Particularly, our ALE analysis supports the *amodal* spatial processing hypothesis (Loomis et al., 2012; Chebat et al., 2018a; Giudice, 2018) as it indicates that EB and SC share common neural networks mediating spatial processing of sensory information, spatial navigation, and the formation of spatial representations. Our data suggest that fronto-parietal networks, typically involved in visuospatial attention and visually guided movements, maintain their function even though spatial information is obtained from sensory modalities other than vision. It is quite unlikely that this can be explained by visual imagery since EB, as defined in this paper, have limited or no visual experience. Furthermore, it is reasonable to assume that scene representations in the PPA is also amodal (Wolbers et al., 2011), as this region is recruited by tactile and auditory information relevant for navigation. However, further evidence is needed to establish the amodal character of the “scene perception” network.

**FIGURE 5:**
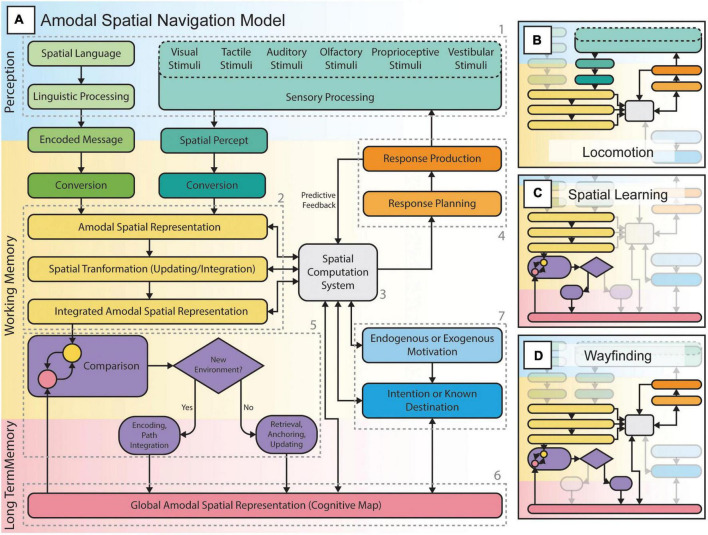
The amodal spatial navigation model including locomotion, spatial learning, and wayfinding. **(A)** Spatial navigation from perception to action, through various spatial computations, planning and execution of responses (actions such as movements and/or attentional allocation to sensory stimuli) according to endogenous or exogenous goals, and the formation and/or use of internal spatial representations (e.g., cognitive maps) to orient oneself in the environment. **(B)** Locomotion or “response-based” navigation consists of a sensorimotor loop that allows the individual to negotiate his path in the environment while considering the presence of obstacles or changes in the ground surface and level. The individual perceives the environment through sensory channels, extracts spatial information and uses it to plan and execute actions. These will then lead to a change (i.e., a new position or viewpoint) in the perceived environment followed by the planning of subsequent actions. This loop is involved in all forms of spatial navigation, including spatial learning and wayfinding tasks. **(C)** Spatial learning is the process of encoding an environment during locomotion (e.g., exploration): the individual integrates various spatial representations and forms a more global cognitive map to be stored in long-term memory. Such spatial representations can either be survey or route knowledge. **(D)** Wayfinding or “cognitive map-based” navigation is the process of retrieving a specific cognitive map and constantly relating it to the perceived environment during locomotion in order to reach known locations while staying oriented in the environment.

In Figure 5, we present a new model of amodal spatial navigation that builds upon the work of various authors (Cisek and Kalaska, 2010; Epstein et al., 2017; Giudice, 2018; Epstein and Baker, 2019). According to this model, amodal spatial representations (2 in Figure 5A), stored in working memory, reflect the external space surrounding the individual (Loomis et al., 2002). These mental representations of space can be formed by sensory experiences and language (1 in Figure 5A), mental imagery or long-term memory, and can persist even after sensory inputs are removed (Giudice, 2018). Through a spatial computation system (3 in Figure 5A), likely the frontoparietal networks identified in the current meta-analysis, amodal spatial representations can serve to plan and execute many types of responses (i.e., locomotion, reaching or eye movements, attentional shifts, etc.; 4 in Figure 5A). According to this model, locomotion (also referred to as egocentric or response-based navigation; Figure 5B), is a sensorimotor loop in which actions lead to new sensory experiences (i.e., a new viewpoint, optic flow, etc.) and, in turn, to new (or updated) spatial representations used to plan subsequent actions. It is also well known that during locomotion, predictive feedback is utilized by the cerebellum to anticipate the consequences of actions and to refine further motor commands (Cisek and Kalaska, 2010; Hull, 2020). According to this model, spatial learning (also referred to as path integration and/or cognitive mapping; Figure 5C) is the process in which amodal spatial representations can be integrated to form a more allocentric (viewpoint-independent) or *global* spatial representation of an environment to be encoded in long-term memory (mediated by the hippocampal complex). These higher-level spatial representations (also known as cognitive maps; 6 in Figure 5A) generally preserve properties and relationships between environmental features such as landmarks, paths and directions (Golledge, 1999; Long and Giudice, 2010). These spatial representations can serve in wayfinding (Figure 5D), as the individual must constantly relate these to perceived features in the environment (5 in Figure 5A) while continuously keeping track of his/her position in relation to those features during locomotion (Long and Giudice, 2010; Epstein et al., 2017; Epstein and Baker, 2019). This constant comparison (5 in Figure 5A) of transient amodal spatial representations (2 in Figure 5A) and long-term global spatial representations (6 in Figure 5A) is likely mediated by the “scene perception” network (Epstein et al., 2017; Epstein and Baker, 2019) and its interaction with frontoparietal networks. This process likely serves different roles during wayfinding: 1) if the individual is in a new environment, a new global spatial representation can be encoded through path integration; 2) if the individual is in a known environment, the global spatial representation can be anchored to the perceived space and, thus, be utilized to orient the individual in this environment. Finally, this model also integrates the individual’s motivations in the processes of wayfinding and locomotion. These motivations can be endogenous (e.g., hunger) or exogenous (e.g., seeing an obstacle or smelling food). These motivations can lead to an intention (e.g., fetch food) or to a known destination (e.g., a restaurant) to make decisions and/or actions (e.g., going to the restaurant). Accordingly, motivations can serve to retrieve global spatial representations and to find a certain destination.

### Navigational abilities in the absence of vision

Spatial navigation involves numerous higher level cognitive processes such as spatial attention, working memory, long-term memory and decision making; consequently, it recruits a large network of brain areas (Epstein et al., 2017; Cona and Scarpazza, 2019; Li et al., 2021). These cognitive processes gain in importance for blind individuals who rely on less precise sensory inputs. Consequently, the formation of spatial representations in blind individuals will require more time and physical exploration of the environment. The difference between blind and sighted individuals is therefore not so much in the ability to form and use spatial representations, but in the temporal aspect of encoding these representations during spatial learning.

Referring to the model of amodal navigation (Figure 5), during locomotion, EB and SC will not only rely on incoming sensory information to guide their movements, but also on the proprioceptive feedback from their own body to judge traveled distances and turns taken. During spatial learning and wayfinding, blind individuals constantly need to memorize this information and keep track of their movements in space as they cannot access all environmental information. Furthermore, wayfinding requires that the individual pays close attention to the limited environmental information they have access to in order to estimate their location in space, to recognize memorized landmarks and other relevant information such as textures on the floor. Consequently, spatial navigation in the absence of vision poses heavier demands on memory and attentional resources which can easily lead to exhaustion (Giudice, 2018). Consequently, many blind individuals tend to limit themselves to familiar environments and routes, which may lead to spatial deficits or delays in the development of spatial and mobility skills during childhood (Millar, 1994; Ungar et al., 1997; Cappagli and Gori, 2016).

**FIGURE 6:**
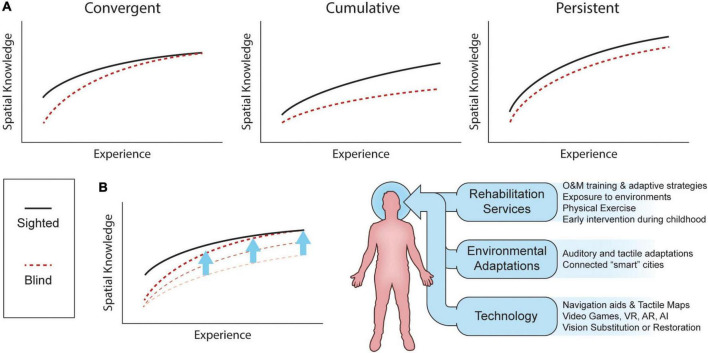
Spatial knowledge acquisition by sighted and blind individuals. **(A)** Three different models of spatial knowledge acquisition: the convergent, cumulative, and persistent models. **(B)** Spatial knowledge acquisition can be improved by the combination of rehabilitation services, environment adaptations and technologies. Adapted, with permission, from Schinazi et al. (2016). AI, artificial intelligence; AR, augmented reality; VR, virtual reality.

There is substantial disagreement as to whether blind individuals possess the same spatial abilities as their sighted counterparts. Figure 6 shows three models of spatial knowledge acquisition in the absence of vision: the *convergent* model, the *deficiency/cumulative* model, and the *inefficiency/persistent* model (reviewed in Schinazi et al., 2016; Giudice, 2018). The *convergent* model suggests that blind subjects are first disadvantaged but that through experience (exposure to an environment, repetition of a task, development of spatial abilities with age) they can reach similar performance as SC. The *cumulative* model proposes that vision plays a critical role in the development of spatial representations and abilities, and that blindness therefore leads to a slower progression in those abilities that will create a disparity between blind and non-blind populations over time. Finally, the *persistent* model suggests that individuals with blindness show a disadvantage from the start that, with age and experience, remains constant as vision is the most effective spatial modality. While firm evidence in support of one of these models is still lacking, novel technologies which aim to substitute or restore vision should bring us as close as possible to performance levels as proposed in the *convergent* model.

While it may be relevant to study and test such models and how tactile and auditory modalities compare to vision for spatial knowledge acquisition, spatial abilities of blind individuals are influenced by numerous internal and external factors. For instance, spatial knowledge and independence vary with age, experience, age at onset of blindness, amount of physical exercise, proficiency with navigational aids (long cane, guide dogs, GPS), the amount of O&M training (use of orientation strategies, practice, echolocation), environmental adaptations and, even, to the specific rehabilitation policies of the country of living. Indeed, O&M specialists often do not have the time to see clients as often as needed to help them develop all spatial skills and concepts that support the formation of allocentric spatial representations of the environment (Giudice, 2018). It is therefore not surprising that there are substantial inter-individual differences in reported spatial performance of blind individuals (Halko et al., 2014; Schinazi et al., 2016). It is hence important to study the numerous factors and neural mechanisms influencing the rate at which spatial abilities can develop and improve in visually impaired subjects of all ages in respect to the degree and onset of their visual condition. For this purpose, virtual environments and videogames show great potential (Connors et al., 2014; Li et al., 2021), but remain largely underused in the field of blindness. Indeed, identifying the neural networks underlying spatial learning and wayfinding in complex, but controlled and low-stress, virtual environments may lead to more adapted rehabilitative strategies and exercises to (1) support better navigational skills and improve autonomy; and (2) develop spatial concepts in children with visual impairments in a way that may decrease developmental delays and cortical atrophy caused by limited experiences and interactions with the environment.

### Study limitations and considerations for future research

The present study is subject to various limitations inherent to the ALE meta-analysis and to working with small study populations. First, the ALE coordinate-based approach is prone to publication bias, false positives, and does not take effect size into account (Müller et al., 2018). Second, studies on blind subjects often deal with recruitment challenges and/or limited sample sizes. Moreover, included blind participants can be very heterogeneous in terms of blindness onset, duration, and cause, factors that may all affect functional outcomes. Many studies did not include LB subjects, implying that this group is underrepresented in the literature. Consequently, the ALE meta-analysis on spatial tasks could only be conducted for EB and SC. Furthermore, very few studies have dealt with the neural mechanisms of navigation, wayfinding and formation of allocentric cognitive maps (Ottink et al., 2022). Future research should therefore focus on better studying these processes; an endeavor that can be facilitated by the recent advances in audio-based virtual reality (Halko et al., 2014; Afonso-Jaco and Katz, 2022; Andrade et al., 2022).

## Conclusion

The present meta-analysis study identified shared neural networks for non-visual spatial processing and navigation in blind and sighted individuals, thus lending further support to the hypothesis stating that neural representations of space are *amodal* or encoded independently of sensory modalities. However, given the limited data on spatial learning and wayfinding in blind populations, future research is still needed to understand their neural correlates. In addition, the paucity of data in late blind subjects also makes it difficult to arrive at firm conclusions regarding the neural correlates of spatial navigation and processing in this group. Since the incidence of late onset blindness is on the rise due to increased longevity and the associated prevalence of age-related diseases and diabetes, it is important to conduct further research in this group of patients.

## Data availability statement

The raw data supporting the conclusions of this article will be made available by the authors, without undue reservation.

## Author contributions

MB, JN, and MP conceptualized the study and planned the experiment. MB planned the methodology. MB, SP, and JN performed the literature search, curated the data, and performed the final formal statistical analysis. MB, SP, D-RC, RK, JN, and MP wrote the original draft, reviewed and edited the manuscript. All authors contributed to the article and approved the submitted version.

## Footnotes

1
http://brainmap.org/ale/

2
http://rii.uthscsa.edu/mango

## References

[B1] Afonso-Jaco A. Katz B. F. (2022). Spatial Knowledge via Auditory Information for Blind Individuals Spatial Cognition Studies and the Use of Audio V R. *Sensors* 22:4794. 10.3390/s22134794 35808291 PMC9268803

[B2] Aguirre G. K. D’esposito M. (1999). Topographical disorientation: A synthesis and taxonomy. *Brain* 122 1613–1628. 10.1093/brain/122.9.1613 10468502

[B3] Andrade R. Baker S. Waycott J. Vetere F. (2022). *A Participatory Design Approach to Creating Echolocation-Enabled Virtual Environments.* New York, NY: Association for Computing Machinery. 10.1145/3516448

[B4] Ankeeta A. Senthil Kumaran S. Saxena R. Dwivedi S. N. Jagannathan N. R. (2021). Visual Cortex Alterations in Early and Late Blind Subjects During Tactile Perception. *Perception* 50 249–265. 10.1177/0301006621991953 33593140

[B5] Anurova I. Renier L. A. De Volder A. G. Carlson S. Rauschecker J. P. (2015). Relationship Between Cortical Thickness and Functional Activation in the Early Blind. *Cereb. Cortex* 25 2035–2048. 10.1093/cercor/bhu009 24518755 PMC4494021

[B6] Arioli M. Ricciardi E. Cattaneo Z. (2021). Social cognition in the blind brain: A coordinate-based meta-analysis. *Hum. Brain Mapp.* 42 1243–1256. 10.1002/hbm.25289 33320395 PMC7927293

[B7] Arnott S. R. Thaler L. Milne J. L. Kish D. Goodale M. A. (2013). Shape-specific activation of occipital cortex in an early blind echolocation expert. *Neuropsychologia* 51 938–949. 10.1016/j.neuropsychologia.2013.01.024 23391560

[B8] Auvray M. Myin E. (2009). Perception with compensatory devices: From sensory substitution to sensorimotor extension. *Cogn. Sci.* 33 1036–1058. 10.1111/j.1551-6709.2009.01040.x 21585495

[B9] Bedny M. Konkle T. Pelphrey K. Saxe R. Pascual-Leone A. (2010). Sensitive Period for a Multimodal Response in Human Visual Motion Area MT/MST. *Curr. Biol.* 20 1900–1906. 10.1016/j.cub.2010.09.044 20970337 PMC2998392

[B10] Bisiach E. Brouchon M. Poncet M. Rusconi M. L. (1993). Unilateral neglect in route description. *Neuropsychologia* 31 1255–1262. 10.1016/0028-3932(93)90072-8 8107985

[B11] Bleau M. Paré S. Djerourou I. Chebat D. R. Kupers R. Ptito M. (2021). Blindness and the Reliability of Downwards Sensors to Avoid Obstacles: A Study with the EyeCane. *Sensors* 21:2700. 10.3390/s21082700 33921202 PMC8070041

[B12] Bonino D. Ricciardi E. Bernardi G. Sani L. Gentili C. Vecchi T. (2015). Spatial imagery relies on a sensory independent, though sensory sensitive, functional organization within the parietal cortex: A fmri study of angle discrimination in sighted and congenitally blind individuals. *Neuropsychologia* 68 59–70. 10.1016/j.neuropsychologia.2015.01.004 25575449

[B13] Bonino D. Ricciardi E. Sani L. Gentili C. Vanello N. Guazzelli M. (2008). Tactile spatial working memory activates the dorsal extrastriate cortical pathway in congenitally blind individuals. *Arch. Ital. Biol.* 146 133–146. 19378878

[B14] Borra E. Luppino G. (2017). Functional anatomy of the macaque temporo-parieto-frontal connectivity. *Cortex* 97 306–326. 10.1016/j.cortex.2016.12.007 28041615

[B15] Burgess N. Becker S. King J. A. O’keefe J. (2001). Memory for events and their spatial context: Models and experiments. *Philos. Trans. R. Soc. Lond. B Biol. Sci.* 356 1493–1503. 10.1098/rstb.2001.0948 11571039 PMC1088531

[B16] Cappagli G. Gori M. (2016). Auditory spatial localization: Developmental delay in children with visual impairments. *Res. Dev. Disabil.* 5 391–398. 10.1016/j.ridd.2016.02.019 27002960

[B17] Cappagli G. Cocchi E. Gori M. (2017). Auditory and proprioceptive spatial impairments in blind children and adults. *Dev. Sci.* 20:e12374. 10.1111/desc.12374 26613827

[B18] Caspers S. Eickhoff S. B. Rick T. Von Kapri A. Kuhlen T. Huang R. (2011). Probabilistic fibre tract analysis of cytoarchitectonically defined human inferior parietal lobule areas reveals similarities to macaques. *NeuroImage* 58 362–380. 10.1016/j.neuroimage.2011.06.027 21718787 PMC8007958

[B19] Cecchetti L. Kupers R. Ptito M. Pietrini P. Ricciardi E. (2016). Are Supramodality and Cross-Modal Plasticity the Yin and Yang of Brain Development? From Blindness to Rehabilitation. *Front. Syst. Neurosci.* 10:89. 10.3389/fnsys.2016.00089 27877116 PMC5099160

[B20] Chan C. C. H. Wong A. W. K. Ting K. H. Whitfield-Gabrieli S. He J. F. Lee T. M. C. (2012). Cross auditory-spatial learning in early-blind individuals. *Hum. Brain Mapp.* 33 2714–2727. 10.1002/hbm.21395 21932260 PMC6870129

[B21] Chebat D. R. Chen J. K. Schneider F. Ptito A. Kupers R. Ptito M. (2007). Alterations in right posterior hippocampus in early blind individuals. *Neuroreport* 18 329–333. 10.1097/WNR.0b013e32802b70f8 17435597

[B22] Chebat D. R. Maidenbaum S. Amedi A. (2015). Navigation Using Sensory Substitution in Real and Virtual Mazes. *PLoS One* 10:e0126307. 10.1371/journal.pone.0126307 26039580 PMC4454637

[B23] Chebat D. R. Maidenbaum S. Amedi A. (2017). “The Transfer of Non-Visual Spatial Knowledge Between Real and Virtual Mazes via Sensory Substitution,” in *2017 International Conference On Virtual Rehabilitation*, (Canada: IEEE). 10.1109/ICVR.2017.8007542

[B24] Chebat D.-R. Schneider F. C. Ptito M. (2020a). Spatial Competence and Brain Plasticity in Congenital Blindness via Sensory Substitution Devices. *Front. Neurosci.* 14:815. 10.3389/fnins.2020.00815 32848575 PMC7406645

[B25] Chebat D. R. Schneider F. C. Ptito M. (2020b). Neural Networks Mediating Perceptual Learning in Congenital Blindness. *Sci. Rep.* 10:495. 10.1038/s41598-019-57217-w 31949207 PMC6965659

[B26] Chebat D.-R. Harrar V. Kupers R. Maidenbaum S. Amedi A. Ptito M. (2018a). “. Sensory Substitution and the Neural Correlates of Navigation in Blindness,” in *Mobility of Visually Impaired People: Fundamentals and ICT Assistive Technologies*, eds Pissaloux E. Velazquez R. (Cham: Springer International Publishing). 10.1007/978-3-319-54446-5_6

[B27] Chebat D.-R. Heimler B. Hofsetter S. Amedi A. (2018b). “The Implications of Brain Plasticity and Task Selectivity for Visual Rehabilitation of Blind and Visually Impaired Individuals,” in *The Neuroimaging of Brain Diseases: Structural and Functional Advances*, ed. Habas C. (Cham: Springer International Publishing). 10.1007/978-3-319-78926-2_13

[B28] Chebat D.-R. Schneider F. C. Kupers R. Ptito M. (2011). Navigation with a sensory substitution device in congenitally blind individuals. *Neuroreport* 22 342–347. 10.1097/WNR.0b013e3283462def 21451425

[B29] Cisek P. Kalaska J. F. (2010). Neural Mechanisms for Interacting with a World Full of Action Choices. *Annu. Rev. Neurosci.* 33 269–298. 10.1146/annurev.neuro.051508.135409 20345247

[B30] Collignon O. Dormal G. Albouy G. Vandewalle G. Voss P. Phillips C. (2013). Impact of blindness onset on the functional organization and the connectivity of the occipital cortex. *Brain* 136 2769–2783. 10.1093/brain/awt176 23831614

[B31] Collignon O. Vandewalle G. Voss P. Albouy G. Charbonneau G. Lassonde M. (2011). Functional specialization for auditory-spatial processing in the occipital cortex of congenitally blind humans. *Proc. Natl. Acad. Sci. U S A.* 108 4435–4440. 10.1073/pnas.1013928108 21368198 PMC3060256

[B32] Collins D. L. Neelin P. Peters T. M. Evans A. C. (1994). Automatic 3D intersubject registration of Mr volumetric data in standardized Talairach space. *J. Comput. Assist. Tomogr.* 18 192–205. 10.1097/00004728-199403000-00005 8126267

[B33] Cona G. Scarpazza C. (2019). Where is the “where” in the brain? A meta-analysis of neuroimaging studies on spatial cognition. *Hum. Brain Mapp.* 40 1867–1886. 10.1002/hbm.24496 30600568 PMC6865398

[B34] Cona G. Semenza C. (2017). Supplementary motor area as key structure for domain-general sequence processing: A unified account. *Neurosci. Biobehav. Rev.* 72 28–42. 10.1016/j.neubiorev.2016.10.033 27856331

[B35] Connors E. C. Chrastil E. R. Sánchez J. Merabet L. B. (2014). Virtual environments for the transfer of navigation skills in the blind: A comparison of directed instruction vs. video game based learning approaches. *Front. Hum. Neurosci.* 8:223. 10.3389/fnhum.2014.00223 24822044 PMC4013463

[B36] Corbetta M. Patel G. Shulman G. L. (2008). The Reorienting System of the Human Brain: From Environment to Theory of Mind. *Neuron* 58 306–324. 10.1016/j.neuron.2008.04.017 18466742 PMC2441869

[B37] Cornette L. Dupont P. Rosier A. Sunaert S. Hecke P. V. Michiels J. (1998). Human brain regions involved in direction discrimination. *J. Neurophysiol.* 79 2749–2765. 10.1152/jn.1998.79.5.2749 9582242

[B38] Deuker L. Bellmund J. L. S. Schröder T. N. Doeller C. F. (2016). An event map of memory space in the hippocampus. *eLife* 5:e16534. 10.7554/eLife.16534 27710766 PMC5053807

[B39] Dodsworth C. (2019). *Changes in the Human Brain Cortex in Response to Learning Click-Based Echolocation: A Virtual Navigation Paradigm*, Ph.D thesis, Durham: Durham University.

[B40] Dormal G. Rezk M. Yakobov E. Lepore F. Collignon O. (2016). Auditory motion in the sighted and blind: Early visual deprivation triggers a large-scale imbalance between auditory and “visual” brain regions. *NeuroImage* 134 630–644. 10.1016/j.neuroimage.2016.04.027 27107468

[B41] Doucet M. E. Guillemot J. P. Lassonde M. Gagné J. P. Leclerc C. Lepore F. (2005). Blind subjects process auditory spectral cues more efficiently than sighted individuals. *Exp. Brain Res.* 160 194–202. 10.1007/s00221-004-2000-4 15309355

[B42] Eickhoff S. B. Bzdok D. Laird A. R. Kurth F. Fox P. T. (2012). Activation likelihood estimation meta-analysis revisited. *NeuroImage* 59 2349–2361. 10.1016/j.neuroimage.2011.09.017 21963913 PMC3254820

[B43] Eickhoff S. B. Bzdok D. Laird A. R. Roski C. Caspers S. Zilles K. (2011). Co-activation patterns distinguish cortical modules, their connectivity and functional differentiation. *NeuroImage* 57 938–949. 10.1016/j.neuroimage.2011.05.021 21609770 PMC3129435

[B44] Eickhoff S. B. Nichols T. E. Laird A. R. Hoffstaedter F. Amunts K. Fox P. T. (2016). Behavior, sensitivity, and power of activation likelihood estimation characterized by massive empirical simulation. *NeuroImage* 137 70–85. 10.1016/j.neuroimage.2016.04.072 27179606 PMC4981641

[B45] Ekstrom A. D. (2015). Why vision is important to how we navigate. *Hippocampus* 25 731–735. 10.1002/hipo.22449 25800632 PMC4449293

[B46] Epstein R. A. (2008). Parahippocampal and retrosplenial contributions to human spatial navigation. *Trends Cogn. Sci.* 12 388–396. 10.1016/j.tics.2008.07.004 18760955 PMC2858632

[B47] Epstein R. A. Baker C. I. (2019). Scene Perception in the Human Brain. *Annu. Rev. Vis. Sci.* 5 373–397. 10.1146/annurev-vision-091718-014809 31226012 PMC6989029

[B48] Epstein R. A. Higgins J. S. (2007). Differential parahippocampal and retrosplenial involvement in three types of visual scene recognition. *Cereb. Cortex* 17, 1680–1693. 16997905 10.1093/cercor/bhl079

[B49] Epstein R. A. Higgins J. S. Jablonski K. Feiler A. M. (2007). Visual scene processing in familiar and unfamiliar environments. *J. Neurophysiol*. 97, 3670–3683. 17376855 10.1152/jn.00003.2007

[B50] Epstein R. A. Patai E. Z. Julian J. B. Spiers H. J. (2017). The cognitive map in humans: Spatial navigation and beyond. *Nat. Neurosci.* 20 1504–1513. 10.1038/nn.4656 29073650 PMC6028313

[B51] Epstein R. Harris A. Stanley D. Kanwisher N. (1999). The parahippocampal place area: Recognition, navigation, or encoding? *Neuron* 23 115–125. 10.1016/S0896-6273(00)80758-8 10402198

[B52] Espinosa M. A. Ungar S. Ochaıìta E. Blades M. Spencer C. (1998). Comparing Methods for Introducing Blind and Visually Impaired People to Unfamiliar Urban Environments. *J. Environ. Psychol.* 18 277–287. 10.1006/jevp.1998.0097

[B53] Farrell M. J. (1996). Topographical disorientation. *Neurocase* 2 509–520. 10.1080/13554799608402427

[B54] Farrell M. J. Robertson I. H. (2000). The automatic updating of egocentric spatial relationships and its impairment due to right posterior cortical lesions. *Neuropsychologia* 38 585–595. 10.1016/S0028-3932(99)00123-2 10689036

[B55] Fiehler K. Burke M. Bien S. Röder B. Rösler F. (2009). The Human Dorsal Action Control System Develops in the Absence of Vision. *Cereb. Cortex* 19 1–12. 10.1093/cercor/bhn067 18448452

[B56] Fiehler K. Schütz I. Meller T. Thaler L. (2015). Neural Correlates of Human Echolocation of Path Direction During Walking. *Multisens. Res.* 28 195–226. 10.1163/22134808-00002491 26152058

[B57] Finocchietti S. Cappagli G. Gori M. (2015). Encoding audio motion: Spatial impairment in early blind individuals. *Front. Psychol.* 6:1357. 10.3389/fpsyg.2015.01357 26441733 PMC4561343

[B58] Fortin M. Voss P. Rainville C. Lassonde M. Lepore F. (2006a). Blind subjects are as good as sighted ones on a topographical orientation task. *Brain Cogn.* 60 309–309.

[B59] Fortin M. Voss P. Rainville C. Lassonde M. Lepore F. (2006b). Impact of vision on the development of topographical orientation abilities. *Neuroreport* 17 443–446. 10.1097/01.wnr.0000203626.47824.86 16514373

[B60] Foulke E. (1982). “Perception, cognition and the mobility of blind pedestrians,” in *Spatial Abilities: Development and Physiological Foundations*, ed. Potegal M. (New York, NY: Academic Press), 55–76.

[B61] Fyhn M. Hafting T. Treves A. Moser M.-B. Moser E. I. (2007). Hippocampal remapping and grid realignment in entorhinal cortex. *Nature* 446 190–194. 10.1038/nature05601 17322902

[B62] Gagnon L. Schneider F. C. Siebner H. R. Paulson O. B. Kupers R. Ptito M. (2012). Activation of the hippocampal complex during tactile maze solving in congenitally blind subjects. *Neuropsychologia* 50 1663–1671. 10.1016/j.neuropsychologia.2012.03.022 22483742

[B63] Garg A. Schwartz D. Stevens A. A. (2007). Orienting auditory spatial attention engages frontal eye fields and medial occipital cortex in congenitally blind humans. *Neuropsychologia* 45 2307–2321. 10.1016/j.neuropsychologia.2007.02.015 17397882 PMC1941615

[B64] Ghaem O. Mellet E. Crivello F. Tzourio N. Mazoyer B. Berthoz A. (1997). Mental navigation along memorized routes activates the hippocampus, precuneus, and insula. *NeuroReport* 8 739–744. 10.1097/00001756-199702100-00032 9106758

[B65] Giudice N. A. (2018). “Navigating without vision: Principles of blind spatial cognition,” in *Handbook of Behavioral and Cognitive Geography*, ed. Montello D. R. (Cheltenham: Edward Elgar Publishing), 432

[B66] Golledge R. G. (1999). *Wayfinding Behavior: Cognitive Mapping and Other Spatial Processes.* Baltimore: John hopkins university press.

[B67] Gori M. Amadeo M. B. Campus C. (2020). Spatial metric in blindness: Behavioural and cortical processing. *Neurosci. Biobehav. Rev.* 109 54–62. 10.1016/j.neubiorev.2019.12.031 31899299

[B68] Gori M. Sandini G. Martinoli C. Burr D. C. (2014). Impairment of auditory spatial localization in congenitally blind human subjects. *Brain* 137 288–293. 10.1093/brain/awt311 24271326 PMC3891446

[B69] Gougoux F. Zatorre R. J. Lassonde M. Voss P. Lepore F. (2005). A Functional Neuroimaging Study of Sound Localization: Visual Cortex Activity Predicts Performance in Early-Blind Individuals. *PLoS Biol.* 3:e27. 10.1371/journal.pbio.0030027 15678166 PMC544927

[B70] Hagler D. J. Sereno M. I. (2006). Spatial maps in frontal and prefrontal cortex. *NeuroImage* 29 567–577. 10.1016/j.neuroimage.2005.08.058 16289928

[B71] Halko M. A. Connors E. C. Sanchez J. Merabet L. B. (2014). Real world navigation independence in the early blind correlates with differential brain activity associated with virtual navigation. *Hum. Brain Mapp.* 35 2768–2778. 10.1002/hbm.22365 24027192 PMC3954447

[B72] Harel A. Kravitz D. J. Baker C. I. (2013). Deconstructing visual scenes in cortex: Gradients of object and spatial layout information. *Cereb. Cortex* 23 947–957. 10.1093/cercor/bhs091 22473894 PMC3593580

[B73] Harrar V. Aubin S. Chebat D.-R. Kupers R. Ptito M. (2018). “The Multisensory Blind Brain,” in *Mobility of Visually Impaired People: Fundamentals and Ict Assistive Technologies*, eds Pissaloux E. Velazquez R. (Cham: Springer International Publishing). 10.1007/978-3-319-54446-5_4

[B74] Hartley T. Harlow R. (2012). An association between human hippocampal volume and topographical memory in healthy young adults. *Front. Hum. Neurosci.* 6:338. 10.3389/fnhum.2012.00338 23293595 PMC3533499

[B75] Hartley T. Maguire E. A. Spiers H. J. Burgess N. (2003). The well-worn route and the path less traveled: Distinct neural bases of route following and wayfinding in humans. *Neuron* 37 877–888. 10.1016/S0896-6273(03)00095-3 12628177

[B76] Hassabis D. Chu C. Rees G. Weiskopf N. Molyneux P. D. Maguire E. A. (2009). Decoding neuronal ensembles in the human hippocampus. *Curr. Biol.* 19 546–554. 10.1016/j.cub.2009.02.033 19285400 PMC2670980

[B77] He C. X. Peelen M. V. Han Z. Z. Lin N. Caramazza A. Bi Y. C. (2013). Selectivity for large nonmanipulable objects in scene-selective visual cortex does not require visual experience. *Neuroimage* 79 1–9. 10.1016/j.neuroimage.2013.04.051 23624496

[B78] Hofstetter S. Zuiderbaan W. Heimler B. Dumoulin S. O. Amedi A. (2021). Topographic maps and neural tuning for sensory substitution dimensions learned in adulthood in a congenital blind subject. *Neuroimage* 235:118029. 10.1016/j.neuroimage.2021.118029 33836269

[B79] Huber E. Jiang F. Fine I. (2019). Responses in area hMT+ reflect tuning for both auditory frequency and motion after blindness early in life. *Proc. Natl. Acad. Sci. U S A.* 116 10081–10086. 10.1073/pnas.1815376116 31036666 PMC6525543

[B80] Hull C. (2020). Prediction signals in the cerebellum: Beyond supervised motor learning. *eLife* 9:e54073. 10.7554/eLife.54073 32223891 PMC7105376

[B81] Iachini T. Ruggiero G. Ruotolo F. (2014). Does blindness affect egocentric and allocentric frames of reference in small and large scale spaces? *Behav. Brain Res.* 273 73–81. 10.1016/j.bbr.2014.07.032 25078290

[B82] Iaria G. Chen J.-K. Guariglia C. Ptito A. Petrides M. (2007). Retrosplenial and hippocampal brain regions in human navigation: Complementary functional contributions to the formation and use of cognitive maps. *Eur. J. Neurosci.* 25 890–899. 10.1111/j.1460-9568.2007.05371.x 17298595

[B83] Iaria G. Petrides M. Dagher A. Pike B. Bohbot V. D. (2003). Cognitive strategies dependent on the hippocampus and caudate nucleus in human navigation: Variability and change with practice. *J. Neurosci. Res.* 23 5945–5952. 10.1523/JNEUROSCI.23-13-05945.2003 12843299 PMC6741255

[B84] Ioannides A. A. Corsi-Cabrera M. Fenwick P. B. C. Del Rio, Portilla Y. Laskaris N. A. (2004). Meg Tomography of Human Cortex and Brainstem Activity in Waking and Rem Sleep Saccades. *Cereb. Cortex* 14 56–72. 10.1093/cercor/bhg091 14654457

[B85] Jalali S. Wohlin C. (2012). *Systematic Literature Studies: Database Searches vs. Backward Snowballing.* Sweden: IEEE. 10.1145/2372251.2372257

[B86] Janzen G. Van Turennout M. (2004). Selective neural representation of objects relevant for navigation. *Nat. Neurosci.* 7 673–677. 10.1038/nn1257 15146191

[B87] Jerde T. A. Curtis C. E. (2013). Maps of space in human frontoparietal cortex. *J. Physiol. Paris* 107 510–516. 10.1016/j.jphysparis.2013.04.002 23603831 PMC3812260

[B88] Jiang A. Tian J. Li R. Liu Y. Jiang T. Qin W. (2015). Alterations of Regional Spontaneous Brain Activity and Gray Matter Volume in the Blind. *Neural Plast.* 2015:141950. 10.1155/2015/141950 26568891 PMC4629052

[B89] Jiang F. Stecker G. C. Boynton G. M. Fine I. (2016). Early Blindness Results in Developmental Plasticity for Auditory Motion Processing within Auditory and Occipital Cortex. *Front. Hum. Neurosci.* 10:324. 10.3389/fnhum.2016.00324 27458357 PMC4932114

[B90] Julian J. B. Ryan J. Epstein R. A. (2017). Coding of object size and object category in human visual cortex. *Cereb. Cortex* 27 3095–3109. 10.1093/cercor/bhw150 27252351 PMC6059234

[B91] Juurmaa J. Suonio K. (1975). The role of audition and motion in the spatial orientation of the blind and the sighted. *Scand. J. Psychol.* 16 209–216. 10.1111/j.1467-9450.1975.tb00185.x 1179180

[B92] Karnath H.-O. Perenin M.-T. (2005). Cortical Control of Visually Guided Reaching: Evidence from Patients with Optic Ataxia. *Cereb. Cortex* 15 1561–1569. 10.1093/cercor/bhi034 15716470

[B93] Kolarik A. J. Pardhan S. Cirstea S. Moore B. C. J. (2017). Auditory spatial representations of the world are compressed in blind humans. *Exp. Brain Res.* 235 597–606. 10.1007/s00221-016-4823-1 27837259 PMC5272902

[B94] Kraeutner S. N. El-Serafi M. Lee J. Boe S. G. (2019). Disruption of motor imagery performance following inhibition of the left inferior parietal lobe. *Neuropsychologia* 127 106–112. 10.1016/j.neuropsychologia.2019.02.016 30807756

[B95] Kupers R. Ptito M. (2014). Compensatory plasticity and cross-modal reorganization following early visual deprivation. *Neurosci. Biobehav. Rev.* 41 36–52. 10.1016/j.neubiorev.2013.08.001 23954750

[B96] Kupers R. Chebat D. R. Madsen K. H. Paulson O. B. Ptito M. (2010). Neural correlates of virtual route recognition in congenital blindness. *Proc. Natl. Acad. Sci. U S A.* 107 12716–12721. 10.1073/pnas.1006199107 20616025 PMC2906580

[B97] Kupers R. Fumal A. De Noordhout A. M. Gjedde A. Schoenen J. Ptito M. (2006). Transcranial magnetic stimulation of the visual cortex induces somatotopically organized qualia in blind subjects. *Proc. Natl. Acad. Sci. U S A.* 103 13256–13260. 10.1073/pnas.0602925103 16916936 PMC1550769

[B98] Lehnert G. Zimmer H. D. (2008). Common coding of auditory and visual spatial information in working memory. *Brain Res*. 1230, 158–167. 18652807 10.1016/j.brainres.2008.07.005

[B99] Lewald J. (2002). Opposing effects of head position on sound localization in blind and sighted human subjects. *Eur. J. Neurosci.* 15 1219–1224. 10.1046/j.1460-9568.2002.01949.x 11982632

[B100] Li J. Zhang R. Liu S. Liang Q. Zheng S. He X. (2021). Human spatial navigation: Neural representations of spatial scales and reference frames obtained from an Ale meta-analysis. *NeuroImage* 238:118264. 10.1016/j.neuroimage.2021.118264 34129948

[B101] Likova L. (2012). The Spatiotopic ‘Visual’ Cortex of the Blind. *Proc. SPIE* 8291:82910L. 10.1117/12.912257

[B102] Linton P. (2021). V1 as an egocentric cognitive map. *Neurosci. Conscious.* 2021:niab017. 10.1093/nc/niab017 34532068 PMC8439394

[B103] Long R. G. Giudice N. A. (2010). “Establishing and Maintaining Orientation for Mobility,” in *Foundations of Orientation and Mobility*, 3 Edn, eds Wiener W. R. Welsh R. L. Blasch B. B. (New York, NY: American Foundation for the Blind.). 10.21307/ijom-2010-008

[B104] Loomis J. M. Klatzky R. L. Giudice N. A. (2013). “Representing 3D Space in Working Memory: Spatial Images from Vision, Hearing, Touch, and Language,” in *Multisensory Imagery*, eds Lacey S. Lawson R. (New York, NY: Springer). 10.1007/978-1-4614-5879-1_8

[B105] Loomis J. M. Klatzky R. L. Golledge R. G. Cicinelli J. G. Pellegrino J. W. Fry P. A. (1993). Nonvisual navigation by blind and sighted: Assessment of path integration ability. *J. Exp. Psychol.* 122:73. 10.1037/0096-3445.122.1.73 8440978

[B106] Loomis J. M. Klatzky R. L. Mchugh B. Giudice N. A. (2012). Spatial working memory for locations specified by vision and audition: Testing the amodality hypothesis. *Atten. Percept. Psychophys.* 74 1260–1267. 10.3758/s13414-012-0311-2 22552825 PMC3482114

[B107] Loomis J. M. Lippa Y. Klatzky R. L. Golledge R. G. (2002). Spatial updating of locations specified by 3-d sound and spatial language. *J. Exp. Psychol. Learn. Mem. Cogn.* 28:335. 10.1037/0278-7393.28.2.335 11911388

[B108] Luna B. Thulborn K. R. Strojwas M. H. Mccurtain B. J. Berman R. A. Genovese C. R. (1998). Dorsal cortical regions subserving visually guided saccades in humans: An fmri study. *Cereb. Cortex* 8 40–47. 10.1093/cercor/8.1.40 9510384

[B109] Mackey W. E. Winawer J. Curtis C. E. (2017). Visual field map clusters in human frontoparietal cortex. *eLife* 6:e22974. 10.7554/eLife.22974 28628004 PMC5491263

[B110] Maidenbaum S. Hanassy S. Abboud S. Buchs G. Chebat D. R. Levy-Tzedek S. (2014). The “EyeCane”, a new electronic travel aid for the blind: Technology, behavior & swift learning. *Restor. Neurol. Neurosci.* 32 813–824. 10.3233/RNN-130351 25201814

[B111] Marchette S. A. Bakker A. Shelton A. L. (2011). Cognitive mappers to creatures of habit: Differential engagement of place and response learning mechanisms predicts human navigational behavior. *J. Neurosci. Res.* 31 15264–15268. 10.1523/JNEUROSCI.3634-11.2011 22031872 PMC4826051

[B112] Matteau I. Kupers R. Ricciardi E. Pietrini P. Ptito M. (2010). Beyond visual, aural and haptic movement perception: HMT+ is activated by electrotactile motion stimulation of the tongue in sighted and in congenitally blind individuals. *Brain Res. Bull.* 82 264–270. 10.1016/j.brainresbull.2010.05.001 20466041

[B113] Merabet L. B. Pascual-Leone A. (2010). Neural reorganization following sensory loss: The opportunity of change. *Nat. Rev. Neurosci.* 11 44–52. 10.1038/nrn2758 19935836 PMC3898172

[B114] Millar S. (1994). *Understanding and Representing Space: Theory and Evidence from Studies with Blind and Sighted Children.* Oxford: Oxford University Press. 10.1093/acprof:oso/9780198521426.001.0001

[B115] Milne J. L. Arnott S. R. Kish D. Goodale M. A. Thaler L. (2015). Parahippocampal cortex is involved in material processing via echoes in blind echolocation experts. *Vis. Res.* 109 139–148. 10.1016/j.visres.2014.07.004 25086210

[B116] Milner D. Goodale M. (2006). *The Visual Brain in Action.* Oxford: Oup Oxford. 10.1093/acprof:oso/9780198524724.001.0001

[B117] Modi S. Bhattacharya M. Singh N. Tripathi R. P. Khushu S. (2012). Effect of visual experience on structural organization of the human brain: A voxel based morphometric study using Dartel. *Eur. J. Radiol.* 81 2811–2819. 10.1016/j.ejrad.2011.10.022 22100371

[B118] Moher D. Liberati A. Tetzlaff J. Altman D. G. (2009). Preferred Reporting Items for Systematic Reviews and Meta-Analyses: The Prisma Statement. *Ann. Intern. Med.* 151 264–269. 10.7326/0003-4819-151-4-200908180-00135 19622511

[B119] Moore T. Fallah M. (2001). Control of eye movements and spatial attention. *Proc. Natl. Acad. Sci. U S A.* 98 1273–1276. 10.1073/pnas.98.3.1273 11158629 PMC14744

[B120] Morgan L. K. Macevoy S. P. Aguirre G. K. Epstein R. A. (2011). Distances between real-world locations are represented in the human hippocampus. *J. Neurosci. Res.* 31 1238–1245. 10.1523/JNEUROSCI.4667-10.2011 21273408 PMC3074276

[B121] Müller F. Niso G. Samiee S. Ptito M. Baillet S. Kupers R. (2019). A thalamocortical pathway for fast rerouting of tactile information to occipital cortex in congenital blindness. *Nat. Commun.* 10:5154. 10.1038/s41467-019-13173-7 31727882 PMC6856176

[B122] Müller V. I. Cieslik E. C. Laird A. R. Fox P. T. Radua J. Mataix-Cols D. (2018). Ten simple rules for neuroimaging meta-analysis. *Neurosci. Biobehav. Rev.* 84 151–161. 10.1016/j.neubiorev.2017.11.012 29180258 PMC5918306

[B123] Myers N. E. Stokes M. G. Nobre A. C. (2017). Prioritizing Information during Working Memory: Beyond Sustained Internal Attention. *Trends Cogn. Sci.* 21 449–461. 10.1016/j.tics.2017.03.010 28454719 PMC7220802

[B124] Nelson J. S. Kuling I. A. Gori M. Postma A. Brenner E. Smeets J. B. J. (2018). Spatial representation of the workspace in blind, low vision, and sighted human participants. *i-Perception* 9:2041669518781877. 10.1177/2041669518781877 29977492 PMC6024533

[B125] Nemmi F. Boccia M. Piccardi L. Galati G. Guariglia C. (2013). Segregation of neural circuits involved in spatial learning in reaching and navigational space. *Neuropsychologia* 51 1561–1570. 10.1016/j.neuropsychologia.2013.03.031 23615031

[B126] Niu M. Rapan L. Funck T. Froudist-Walsh S. Zhao L. Zilles K. (2021). Organization of the macaque monkey inferior parietal lobule based on multimodal receptor architectonics. *NeuroImage* 231:117843. 10.1016/j.neuroimage.2021.117843 33577936 PMC8188735

[B127] Norman L. J. Thaler L. (2019). Retinotopic-like maps of spatial sound in primary ‘visual’ cortex of blind human echolocators. *Proc. Biol. Sci.* 286:20191910. 10.1098/rspb.2019.1910 31575359 PMC6790759

[B128] Ohnishi T. Matsuda H. Hirakata M. Ugawa Y. (2006). Navigation ability dependent neural activation in the human brain: An fMRI study. *Neurosci. Res.* 55 361–369. 10.1016/j.neures.2006.04.009 16735070

[B129] Ottink L. Buimer H. Van Raalte B. Doeller C. F. Van Der Geest T. M. Van Wezel R. J. A. (2022). Cognitive map formation supported by auditory, haptic, and multimodal information in persons with blindness. *Neurosci. Biobehav. Rev.* 140:104797. 10.1016/j.neubiorev.2022.104797 35902045

[B130] Palejwala A. H. Dadario N. B. Young I. M. O’connor K. Briggs R. G. Conner A. K. (2021). Anatomy and white matter connections of the lingual gyrus and cuneus. *World Neurosurg.* 151 e426–e437. 10.1016/j.wneu.2021.04.050 33894399

[B131] Paré S. Bleau M. Djerourou I. Malotaux V. Kupers R. Ptito M. (2021). Spatial navigation with horizontally spatialized sounds in early and late blind individuals. *PLoS One* 16:e0247448. 10.1371/journal.pone.0247448 33635892 PMC7909643

[B132] Park H.-J. Chun J.-W. Park B. Park H. Kim J. I. Lee J. D. (2011). Activation of the Occipital Cortex and Deactivation of the Default Mode Network During Working Memory in the Early Blind. *J. Int. Neuropsychol. Soc.* 17 407–422. 10.1017/S1355617711000051 21338547

[B133] Park S. Chun M. M. (2009). Different roles of the parahippocampal place area (Ppa) and retrosplenial cortex (Rsc) in panoramic scene perception. *NeuroImage* 47 1747–1756. 10.1016/j.neuroimage.2009.04.058 19398014 PMC2753672

[B134] Parlatini V. Radua J. Dell’acqua F. Leslie A. Simmons A. Murphy D. G. (2017). Functional segregation and integration within fronto-parietal networks. *NeuroImage* 146 367–375. 10.1016/j.neuroimage.2016.08.031 27639357 PMC5312783

[B135] Pasqualotto A. Newell F. N. (2007). The role of visual experience on the representation and updating of novel haptic scenes. *Brain Cogn.* 65 184–194. 10.1016/j.bandc.2007.07.009 17845829

[B136] Pasqualotto A. Proulx M. J. (2012). The role of visual experience for the neural basis of spatial cognition. *Neurosci. Biobehav. Rev.* 36 1179–1187. 10.1016/j.neubiorev.2012.01.008 22330729

[B137] Passini R. Proulx G. (1988). Wayfinding without Vision: An Experiment with Congenitally Totally Blind People. *Environ. Behav.* 20 227–252. 10.1177/0013916588202006

[B138] Passini R. Proulx G. Rainville C. (1990). The spatio-cognitive abilities of the visually impaired population. *Environ. Behav.* 22 91–118. 10.1177/0013916590221005

[B139] Paus T. (1996). Location and function of the human frontal eye-field: A selective review. *Neuropsychologia* 34 475–483. 10.1016/0028-3932(95)00134-4 8736560

[B140] Pick H. L. (1974). “Visual coding of nonvisual spatial information,” in *Perception: Essays in honor of James J. Gibson*, eds MacLeod R. B. Pickeds H. L. (Ithaca, NY: Cornell University Press).

[B141] Power, Jonathan D. Cohen, Alexander L. Nelson, Steven M. (2011). Functional Network Organization of the Human Brain. *Neuron* 72 665–678. 10.1016/j.neuron.2011.09.006 22099467 PMC3222858

[B142] Prado J. Clavagnier S. Otzenberger H. Scheiber C. Kennedy H. Perenin M.-T. (2005). Two Cortical Systems for Reaching in Central and Peripheral Vision. *Neuron* 48 849–858. 10.1016/j.neuron.2005.10.010 16337921

[B143] Proulx M. J. Brown D. J. Pasqualotto A. Meijer P. (2014). Multisensory perceptual learning and sensory substitution. *Neurosci. Biobehav. Rev.* 41 16–25. 10.1016/j.neubiorev.2012.11.017 23220697

[B144] Ptito M. Kupers R. (2005). Cross-modal plasticity in early blindness. *J. Integr. Neurosci.* 4 479–488. 10.1142/S0219635205000951 16385642

[B145] Ptito M. Bleau M. Djerourou I. Paré S. Schneider F. C. Chebat D.-R. (2021a). Brain-Machine Interfaces to Assist the Blind. *Front. Hum. Neurosci.* 15:46388876. 10.3389/fnhum.2021.638887 33633557 PMC7901898

[B146] Ptito M. Fumal A. De Noordhout A. M. Schoenen J. Gjedde A. Kupers R. (2008a). Tms of the occipital cortex induces tactile sensations in the fingers of blind Braille readers. *Exp. Brain Res.* 184 193–200. 10.1007/s00221-007-1091-0 17717652

[B147] Ptito M. Matteau I. Gjedde A. Kupers R. (2009). Recruitment of the middle temporal area by tactile motion in congenital blindness. *NeuroReport* 20 543–547. 10.1097/WNR.0b013e3283279909 19240660

[B148] Ptito M. Moesgaard S. M. Gjedde A. Kupers R. (2005). Cross-modal plasticity revealed by electrotactile stimulation of the tongue in the congenitally blind. *Brain* 128 606–614. 10.1093/brain/awh380 15634727

[B149] Ptito M. Paré S. Dricot L. Cavaliere C. Tomaiuolo F. Kupers R. (2021b). A quantitative analysis of the retinofugal projections in congenital and late-onset blindness. *NeuroImage* 32:102809. 10.1016/j.nicl.2021.102809 34509923 PMC8435915

[B150] Ptito M. Schneider F. C. G. Paulson O. B. Kupers R. (2008b). Alterations of the visual pathways in congenital blindness. *Exp. Brain Res.* 187 41–49. 10.1007/s00221-008-1273-4 18224306

[B151] Qiu Y. Wu Y. Liu R. Wang J. Huang H. Huang R. (2019). Representation of human spatial navigation responding to input spatial information and output navigational strategies: An ALE meta-analysis. *Neurosci. Biobehav. Rev.* 103 60–72. 10.1016/j.neubiorev.2019.06.012 31201830

[B152] Reislev N. L. Kupers R. Siebner H. R. Ptito M. Dyrby T. B. (2016). Blindness alters the microstructure of the ventral but not the dorsal visual stream. *Brain Struct. Funct.* 221 2891–2903. 10.1007/s00429-015-1078-8 26134685

[B153] Renier L. A. Anurova I. De Volder A. G. Carlson S. Vanmeter J. Rauschecker J. P. (2010). Preserved functional specialization for spatial processing in the middle occipital gyrus of the early blind. *Neuron* 68 138–148. 10.1016/j.neuron.2010.09.021 20920797 PMC2951740

[B154] Ricciardi E. Tozzi L. Leo A. Pietrini P. (2014). Modality Dependent Cross-Modal Functional Reorganization Following Congenital Visual Deprivation within Occipital Areas: A Meta-Analysis of Tactile and Auditory Studies. *Multisensory Res.* 27 247–262. 10.1163/22134808-00002454 25577905

[B155] Ricciardi E. Vanello N. Sani L. Gentili C. Scilingo E. P. Landini L. (2007). The effect of visual experience on the development of functional architecture in hMT. *Cereb. Cortex* 17 2933–2939. 10.1093/cercor/bhm018 17372275

[B156] Rogge A. K. Hamacher D. Cappagli G. Kuhne L. Hötting K. Zech A. (2021). Balance, gait, and navigation performance are related to physical exercise in blind and visually impaired children and adolescents. *Exp. Brain Res.* 239 1111–1123. 10.1007/s00221-021-06038-3 33550429 PMC8068618

[B157] Ruggiero G. Ruotolo F. Iachini T. (2018). Congenital blindness limits allocentric to egocentric switching ability. *Exp. Brain Res.* 236 813–820. 10.1007/s00221-018-5176-8 29340716

[B158] Ruggiero G. Ruotolo F. Iachini T. (2021). How ageing and blindness affect egocentric and allocentric spatial memory. *Q. J. Exp. Psychol.* 75 1628–1642. 10.1177/17470218211056772 34670454

[B159] Saygin A. P. Sereno M. I. (2008). Retinotopy and Attention in Human Occipital Temporal, Parietal, and Frontal Cortex. *Cereb. Cortex* 18 2158–2168. 10.1093/cercor/bhm242 18234687

[B160] Schinazi V. R. Epstein R. A. (2010). Neural correlates of real-world route learning. *Neuroimage* 53 725–735. 10.1016/j.neuroimage.2010.06.065 20603219

[B161] Schinazi V. R. Nardi D. Newcombe N. S. Shipley T. F. Epstein R. A. (2013). Hippocampal size predicts rapid learning of a cognitive map in humans. *Hippocampus* 23 515–528. 10.1002/hipo.22111 23505031 PMC3690629

[B162] Schinazi V. R. Thrash T. Chebat D.-R. (2016). Spatial navigation by congenitally blind individuals. *Wires Cogn. Sci.* 7 37–58. 10.1002/wcs.1375 26683114 PMC4737291

[B163] Schindler A. Bartels A. (2013). Parietal Cortex Codes for Egocentric Space beyond the Field of View. *Curr. Biol.* 23 177–182. 10.1016/j.cub.2012.11.060 23260468

[B164] Seeley W. W. Menon V. Schatzberg A. F. Keller J. Glover G. H. Kenna H. (2007). Dissociable Intrinsic Connectivity Networks for Salience Processing and Executive Control. *J. Neurosci.* 27:2349. 10.1523/JNEUROSCI.5587-06.2007 17329432 PMC2680293

[B165] Segond H. Weiss D. Sampaio E. (2005). Human spatial navigation via a visuo-tactile sensory substitution system. *Perception* 34 1231–1249. 10.1068/p3409 16309117

[B166] Serences J. T. Yantis S. (2007). Spatially Selective Representations of Voluntary and Stimulus-Driven Attentional Priority in Human Occipital Parietal, and Frontal Cortex. *Cereb. Cortex* 17 284–293. 10.1093/cercor/bhj146 16514108

[B167] Shimony J. S. Burton H. Epstein A. A. Mclaren D. G. Sun S. W. Snyder A. Z. (2006). Diffusion Tensor Imaging Reveals White Matter Reorganization in Early Blind Humans. *Cereb. Cortex* 16 1653–1661. 10.1093/cercor/bhj102 16400157 PMC3789517

[B168] Silva P. R. Farias T. Cascio F. Dos Santos L. Peixoto V. Crespo E. (2018). Neuroplasticity in Visual Impairments. *Neurol. Int.* 10:7326. 10.4081/ni.2018.7326 30687464 PMC6322049

[B169] Silver M. A. Ress D. Heeger D. J. (2005). Topographic Maps of Visual Spatial Attention in Human Parietal Cortex. *J. Neurophysiol.* 94 1358–1371. 10.1152/jn.01316.2004 15817643 PMC2367310

[B170] Singh-Curry V. Husain M. (2009). The functional role of the inferior parietal lobe in the dorsal and ventral stream dichotomy. *Neuropsychologia* 47 1434–1448. 10.1016/j.neuropsychologia.2008.11.033 19138694 PMC2697316

[B171] Spiers H. J. Maguire E. A. (2007). Decoding human brain activity during real-world experiences. *Trends Cogn. Sci.* 11 356–365. 10.1016/j.tics.2007.06.002 17618161

[B172] Squire R. F. Noudoost B. Schafer R. J. Moore T. (2013). Prefrontal Contributions to Visual Selective Attention. *Annu. Rev. Neurosci.* 36 451–466. 10.1146/annurev-neuro-062111-150439 23841841

[B173] Stilla R. Hanna R. Hu X. P. Mariola E. Deshpande G. Sathian K. (2008). Neural processing underlying tactile microspatial discrimination in the blind: A functional magnetic resonance imaging study. *J. Vis.* 8:13. 10.1167/8.10.13 PMC3063091 19146355

[B174] Stoll C. Palluel-Germain R. Fristot V. Pellerin D. Alleysson D. Graff C. (2015). Navigating from a depth image converted into sound. *Appl. Bionics. Biomech.* 2015:543492. 10.1155/2015/543492 27019586 PMC4745448

[B175] Striem-Amit E. Dakwar O. Reich L. Amedi A. (2012). The large-Scale Organization of Visual Streams Emerges Without Visual Experience. *Cereb. Cortex* 22 1698–1709. 10.1093/cercor/bhr253 21940707

[B176] Struiksma M. E. Noordzij M. L. Neggers S. F. Bosker W. M. Postma A. (2011). Spatial language processing in the blind: Evidence for a supramodal representation and cortical reorganization. *PLoS One* 6:e24253. 10.1371/journal.pone.0024253 21935391 PMC3173383

[B177] Sun J. Huang J. Wang A. Zhang M. Tang X. (2022). The role of the interaction between the inferior parietal lobule and superior temporal gyrus in the multisensory Go/No-go task. *NeuroImage* 254:119140. 10.1016/j.neuroimage.2022.119140 35342002

[B178] Suthana N. A. Ekstrom A. D. Moshirvaziri S. Knowlton B. Bookheimer S. Y. (2009). Human hippocampal CA1 involvement during allocentric encoding of spatial information. *J. Neurosci.* 29 10512–10519. 10.1523/JNEUROSCI.0621-09.2009 19710304 PMC2873654

[B179] Talairach J. Tournoux P. (1988). *Co-Planar Stereotaxic Atlas of the Human Brain.* New York, NY: Thieme.

[B180] Tao Q. Chan C. C. H. Luo Y. J. Li J. J. Ting K. H. Lu Z. L. (2017). Prior Visual Experience Modulates Learning of Sound Localization Among Blind Individuals. *Brain Topography* 30 364–379. 10.1007/s10548-017-0549-z 28161728 PMC5408050

[B181] Tao Q. Chan C. C. H. Luo Y.-J. Li J.-J. Ting K.-H. Wang J. (2015). How Does Experience Modulate Auditory Spatial Processing in Individuals with Blindness? *Brain Topography* 28 506–519. 10.1007/s10548-013-0339-1 24322827 PMC4408360

[B182] Teng S. Puri A. Whitney D. (2012). Ultrafine spatial acuity of blind expert human echolocators. *Exp. Brain Res.* 216 483–488. 10.1007/s00221-011-2951-1 22101568 PMC3849401

[B183] Thaler L. Arnott S. R. Goodale M. A. (2011). Neural correlates of natural human echolocation in early and late blind echolocation experts. *PLoS One* 6:e20162. 10.1371/journal.pone.0020162 21633496 PMC3102086

[B184] Thinus-Blanc C. Gaunet F. (1997). Representation of space in blind persons: Vision as a spatial sense? *Psychol. Bull.* 121 20–42. 10.1037/0033-2909.121.1.20 9064698

[B185] Turkeltaub P. E. Eickhoff S. B. Laird A. R. Fox M. Wiener M. Fox P. (2012). Minimizing within-experiment and within-group effects in activation likelihood estimation meta-analyses. *Hum. Brain Mapp.* 33 1–13. 10.1002/hbm.21186 21305667 PMC4791073

[B186] Ungar S. Blades M. Spencer C. (1997). Teaching visually impaired children to make distance judgments from a tactile map. *J. Vis. Impair. Blind.* 91 163–174. 10.1177/0145482X9709100209

[B187] Van der Heijden K. Formisano E. Valente G. Zhan M. Kupers R. De Gelder B. (2020). Reorganization of Sound Location Processing in the Auditory Cortex of Blind Humans. *Cereb. Cortex* 30 1103–1116. 10.1093/cercor/bhz151 31504283

[B188] Vandenberghe R. Molenberghs P. Gillebert C. R. (2012). Spatial attention deficits in humans: The critical role of superior compared to inferior parietal lesions. *Neuropsychologia* 50 1092–1103. 10.1016/j.neuropsychologia.2011.12.016 22266260

[B189] Vernet M. Quentin R. Chanes L. Mitsumasu A. Valero-Cabré A. (2014). Frontal eye field, where art thou? Anatomy, function, and non-invasive manipulation of frontal regions involved in eye movements and associated cognitive operations. *Front. Integr. Neurosci.* 8:66. 10.3389/fnint.2014.00066 25202241 PMC4141567

[B190] Voss P. Gougoux F. Lassonde M. Zatorre R. J. Lepore F. (2006). A positron emission tomography study during auditory localization by late-onset blind individuals. *NeuroReport* 17 383–388. 10.1097/01.wnr.0000204983.21748.2d 16514363

[B191] Voss P. Gougoux F. Zatorre R. J. Lassonde M. Lepore F. (2008). Differential occipital responses in early- and late-blind individuals during a sound-source discrimination task. *NeuroImage* 40 746–758. 10.1016/j.neuroimage.2007.12.020 18234523

[B192] Voss P. Lassonde M. Gougoux F. Fortin M. Guillemot J. P. Lepore F. (2004). Early- and late-onset blind individuals show supra-normal auditory abilities in far-space. *Curr. Biol.* 14 1734–1738. 10.1016/j.cub.2004.09.051 15458644

[B193] Voss P. Lepore F. Gougoux F. Zatorre R. J. (2011). Relevance of spectral cues for auditory spatial processing in the occipital cortex of the blind. *Front. Psychol.* 2:48. 10.3389/fpsyg.2011.00048 21716600 PMC3110881

[B194] Wang D. Qin W. Liu Y. Zhang Y. Jiang T. Yu C. (2013). Altered White Matter Integrity in the Congenital and Late Blind People. *Neural Plast.* 2013:128236. 10.1155/2013/128236 23710371 PMC3654351

[B195] Weeks R. Horwitz B. Aziz-Sultan A. Tian B. Wessinger C. M. Cohen L. G. (2000). A positron emission tomographic study of auditory localization in the congenitally blind. *J. Neurosci.* 20 2664–2672. 10.1523/JNEUROSCI.20-07-02664.2000 10729347 PMC6772250

[B196] Wittenberg G. F. Werhahn K. J. Wassermann E. M. Herscovitch P. Cohen L. G. (2004). Functional connectivity between somatosensory and visual cortex in early blind humans. *Eur. J. Neurosci.* 20 1923–1927. 10.1111/j.1460-9568.2004.03630.x 15380014

[B197] Wolbers T. Büchel C. (2005). Dissociable retrosplenial and hippocampal contributions to successful formation of survey representations. *J. Neurosci.* 25 3333–3340. 10.1523/JNEUROSCI.4705-04.2005 15800188 PMC6724902

[B198] Wolbers T. Klatzky R. L. Loomis J. M. Wutte M. G. Giudice N. A. (2011). Modality-independent coding of spatial layout in the human brain. *Curr. Biol.* 21 984–989. 10.1016/j.cub.2011.04.038 21620708 PMC3119034

[B199] Woollett K. Maguire E. A. (2011). Acquiring “the Knowledge” of London’s layout drives structural brain changes. *Curr. Biol.* 21 2109–2114. 10.1016/j.cub.2011.11.018 22169537 PMC3268356

[B200] Wu Y. Wang J. Zhang Y. Zheng D. Zhang J. Rong M. (2016). The Neuroanatomical Basis for Posterior Superior Parietal Lobule Control Lateralization of Visuospatial Attention. *Front. Neuroanat.* 10:32. 10.3389/fnana.2016.00032 27047351 PMC4805595

[B201] Zhang C. Lee T. M. C. Fu Y. Ren C. Chan C. C. H. Tao Q. (2019). Properties of cross-modal occipital responses in early blindness: An Ale meta-analysis. *NeuroImage* 24:102041. 10.1016/j.nicl.2019.102041 31677587 PMC6838549

[B202] Zwiers M. P. Van Opstal A. J. Cruysberg J. R. (2001). A spatial hearing deficit in early-blind humans. *J. Neurosci.* 21:RC142. 10.1523/JNEUROSCI.21-09-j0002.2001 11312316 PMC6762556

