# Supplementary material for: Neural substrates of spatial processing and navigation in blindness: An activation likelihood estimation meta-analysis
Source: Front Neurosci. 2022 Oct 20;16:1010354. doi: 10.3389/fnins.2022.1010354 (PMC9630591; doi:10.3389/fnins.2022.1010354)
Supplement: Supplementary file 1 [file Data_Sheet_1.docx]

**Table S1. Keywords used in the literature search.**

| Concept | Keywords |
| --- | --- |
| Spatial cognition and navigation | "Space" OR "Spatial" OR "Spatial cognition" OR "Spatial representation" OR "Spatial process" OR "Spatial skill" OR "Spatial memory" OR "Spatial thinking" OR "Spatial awareness" OR "Localization" OR "Navigation" OR "Navigating" OR "Locomotion" OR "Orientation" OR "Orienting" OR "Reorientation" OR "Wayfinding" OR "Path integration" OR "Cognitive map" OR "Mental map" OR "Cognitive mapping" OR "Spatial learning" OR "Spatial knowledge" OR "Survey knowledge" OR "Spatial layout" OR "Route learning" OR "Reference frames" OR "Egocentric–allocentric reference frames" OR "Allocentric" OR "Route" OR "Maze" OR "Path" OR "Place" OR "Map" OR "Position" OR "Direction" OR "Travel" |
| Neuroimagery | "fMRI" OR "PET" OR "Neural correlates" OR "Neural substrates" OR "Brain" OR "Cortical" OR "Cerebral" OR "Plasticity" OR "Brain imaging" OR "Cortical areas" OR "Cortical activation" OR "BOLD signal" |
| Blindness | "Blind" OR "Blindness" OR "Vision impairment" OR "Visual impairment" OR "Visually impaired" OR "Without vision" OR "Non-visual" OR "Early blind" OR "Congenital blind" OR "Congenitally blind" OR "Congenital blindness" OR "Early onset blindness" OR "Adventitiously blind" OR "Late blind" OR "Late onset blindness" |

**Table S2. Characteristics and main findings of sources included in the qualitative synthesis but excluded for ALE meta-analysis.**

| Reference | Functional domain | Method | N subjects | Reason for ALE exclusion | Task contrasts | Main findings |
| --- | --- | --- | --- | --- | --- | --- |
| Anurova et al. (2019) | Auditory localization, working memory | fMRI | 15 EB, 15 SC | ROI | Sound localization (one-back) > baseline | Occipital areas (calcarine sulcus and lateral occipital) are cross-modally deactivated in SC but activated (with auditory cortex) in EB. Interactions between frontal cortex and MDN of thalamus seem essential for controlling temporal and occipital cortex activity during auditory working memory. |
| Arnott et al. (2013) | Active echolocation | fMRI | 2 EB, 1 LB, 3 SC | Less than three participants per group | Shape > material; material > shape; place > material & shape | Echolocation-related activations in the V1 of EB are topographically organized in the same way as visual related activations in SC: by angle and eccentricity (center = occipital pole, periphery = more rostral), while contralateral representations are also preserved. |
| Collignon et al. (2013) | Auditory localization | fMRI | 12 EB, 10 LB, 22 SC | Coordinates not reported | Spatial judgment > pitch judgment | LB and EB recruited the same occipital areas, but cuneus activity was higher in EB. Only EB showed spatial selectivity in right MOG, SOG, middle occipitotemporal gyrus, and cuneus. These areas were functionally connected to IPS. Dynamic causal modelling revealed potentially different connectivity architecture: EB = A1 to V1 to IPS; LB = A1 to IPS to V1. |
| De Volder et al. (1999) | Object localization with SSD | PET | 5 EB, 4 SC | Coordinates not reported | Localization of silent objects with ssd > localization of sounds | More glucose activity in the occipital cortex of EB. After training with SSD, occipital areas (BA 7, 18, 19 bilaterally, and BA 22 in right hemisphere) were more recruited when locating objects with the SSD than when locating real sounds. |
| Garg et al. (2007) | Auditory spatial attention | fMRI | 9 EB, 10 SC | ROI | Covert attention switching > rest | Auditory covert attention activated FEF in both EB and SC, but its activity in auditory modality is higher in EB. EB also activated early visual areas (medial occipital including calcarine sulcus). |
| Goyal et al. (2006) | Tactile motion processing | fMRI | 3 EB, 3 LB, 3 SC | ROI | Moving stimuli > static stimuli | During tactile motion perception (stimuli passively presented on the palm), only LB exhibited activations in the region of interest: hMT/V5. |
| Hofstetter et al. (2021) | Spatial representations with SSD | fMRI | 1 EB | Less than three participants per group | Moving visual-to-auditory stimuli > no stimuli | Topographic maps of 2D soundspace (x=time, y=pitch) are found in the right occipito-temporal cortex (lateral occipital) and parietal cortex (superior PPC, SMG) suggesting a mapping like topographic organization of retinotopic positions in visual space. |
| Huber et al. (2019) | Auditory motion processing | fMRI | 4 EB, 6 SC | ROI | Moving stimuli > static stimuli | The distribution of frequencies did not differ between hMT+ and A1 in all groups. Tuning widths in hMT+ in eb and sc were similar between hMT+ and primary auditory cortex. Evidence for frequency tuning in hMT+ was found for SC and only in 1 EB participant. |
| Jiang et al. (2014) | Auditory motion processing | fMRI | 7 EB, 7 SC | ROI | Unambiguous (moving) stimuli > ambiguous (no motion) stimuli | EB showed superior activation in hMT+ and in LOC. No difference was found in planum temporal and A1. In EB, unambiguous stimuli affected hMT+ bilaterally whereas ambiguous stimuli only affected the left hMT+. In SC, there were no effect of stimulus type, nor differences between cerebral hemispheres. |
| Jiang et al. (2016) | Auditory motion processing | fMRI | 7 EB, 4 LB, 11 SC | ROI | Unambiguous (moving) stimuli > ambiguous (no motion) stimuli | EB showed superior activation in hMT+ and in LOC. No difference was found in planum temporal and A1. In EB, unambiguous stimuli affected hMT+ bilaterally whereas ambiguous stimuli only affected the left hMT+. In SC, there were no effect of stimulus type, nor differences between cerebral hemispheres. |
| Likova (2010) | Tactile memory drawing | fMRI | 9 subjects (blind and SC) | Coordinates not reported | Tactilely guided copy (drawing) & tactile exploration > scribble motor control | EB and SC showed similar cortical networks. EB activated, to a larger extent than SC, occipital areas (lingual gyrus and cuneus), MTG, SMG, somatosensory regions, SPL, IPS, primary motor and premotor areas. LOC (object-recognition) had strongest activations during exploration, while V1 and visual motion areas (hMT, hMT+, hMST) were equally activated during exploration and drawing in EB. These occipital areas were suppressed in SC. |
| Likova (2012) | Tactile memory drawing | fMRI | 6 EB, 7 LB | ROI, coordinates not reported | Tactile memory drawing > scribble motor control | Activations of V1 in all groups (EB, LB and blindfolded SC from a previous study) during drawing expanded to the same eccentricity (10 degrees) and thus might suggest a similar “spatiotopic” representation within V1 that might be independent from vision. |
| Lewis et al. (2010) | Auditory motion processing, tactile orientation discrimination | fMRI | 7 EB, 6 SC | ROI | Auditory motion > motor control; tactile orientation > motor control | In EB, auditory motion elicited cross-modal activations in all occipital rois including foveal and peripheral early visual areas (V1, V2), lateral occipital sulcus, V3, V4, hMT+, and early IPS. As of tactile orientation, all ROIs were also activated except the foveal confluence. In SC, auditory motion and tactile orientation was associated with occipital and occipito-parietal deactivations. |
| Maidenbaum et al. (2018) | Virtual navigation with simulated auditory ssd (eyecane) | fMRI | 10 EB, 9 SC | ROI | Maze navigation > rest or scramble; post-training maze navigation > pre-training maze navigation | V6/V6a (dorsal precuneus) was activated in SC (blindfolded and eyes open) and EB during navigation in mazes. After training with the SSD, navigation selectively recruited peripheral V1 in EB and peripheral and foveal V1 in SC. Activations were also found in the striatum and cortical motor regions, while deactivations were found in the posterior hippocampus. |
| Norman and Thaler (2019) | Echolocation | fMRI | 6 EB, 4 LB, 5 SC | No univariate approach | Stimuli (echo recordings, source sounds or visual stimuli) localization > baseline (no stimuli) | Via eccentricity mapping of echo sounds, source sounds and visual stimuli, authors found that in V1, stimulus maps for sound in expert echolocators are similar to stimulus maps for vision in SC. This similarity is corelated with echolocation ability. This result hints towards a “task-specific” organization in primary sensory cortices. |
| Poirier et al. (2006) | Auditory motion processing | fMRI | 6 EB, 6 SC | ROI | Motion > static stimuli; motion > rest | Both groups showed significant bilateral activations in the dorsal and ventral premotor cortex, V3/V3a and V1/V2, as well as activations in the left IPL and right V5. Activations in the left ventral premotor cortex, bilateral V3/V3a and bilateral V1/V2 were higher in EB. Occipital activations (V1, V2, V5, V3/V3a) were significantly higher for motion stimuli than static stimuli. |
| (Strnad et al., 2013) | Auditory motion processing | fMRI | 10 EB, 20 SC | ROI | High motion > low motion; motion > rest | Univariate approach revealed that hMT+ is only responsive to auditory motion in EB. Multi-voxel pattern analysis, however, revealed no difference in hMT+ activity difference between groups, thus suggesting that information about auditory motion is present in hMT+ in both EB and SC. |
| Thaler et al. (2011) | Echolocation | fMRI | 1 EB, 1 LB, 2 SC | Less than three participants per group | Recordings with echoes > recordings without echoes; moving targets > static targets | EB and LB, as expert echolocators, activated calcarine cortex to process click echoes reflecting on targets. This activity was higher in EB and showed bias towards processing echoes from the contralateral space (like the calcarine cortex in SC does for light). Medial frontal sulcus and cerebellum activations were also found in EB and LB. hMT+ was also recruited to process echoes from moving targets. |
| Thaler et al. (2014) | Auditory motion processing, echolocation | fMRI | 2 EB, 1 LB, 12 SC | Coordinates not reported | Moving target (echo sound, source sound, visual) > stationary; left > right | There was a double dissociation between echo-motion and source-motion processing in both blind and SC groups: temporal-occipital source-motion ROIs did not respond to echo-motion, and echo-motion ROIs did not respond to source-motion. Blind temporal-occipital echo-motion ROIs, but not source-motion ROIs, demonstrated contralateral motion preference. |
| van der Heijden et al. (2020) | Auditory localization | fMRI | 12 EB, 12 SC | Coordinates not reported | Target (auditory or visual) presentation > no stimuli | Compared to SC, EB showed stronger activation of the occipital cortex (cuneus, calcarine and posterior occipital sulci), but reduced activation in the medial temporal cortex. Activation patterns for binaural spatial processing differed between the two groups. The auditory cortex of EB also carried less information on sound azimuth position than SC. |
| Wallmeier et al. (2015) | Echolocation | fMRI | 2 EB, 4 SC | Less than three participants per group | Localization (single reflector) > differentiation (single vs dual) | Calcarine cortex is strongly activated in EB during echolocation. Its activation pattern could discriminate left-side from right-side stimuli, which was corelated with echolocation ability. While there were no occipital activations in SC, activation patterns in the planum temporale could discriminate left-side from right-side stimuli. |
| Wolbers et al. (2011) | Auditory motion processing | fMRI | 11 EB, 9 SC | ROI | Moving sound > stationary sound; left-ward sounds > right-ward sounds | In EB, auditory motion evoked directional responses in dorsal occipito-temporal areas encompassing the hMT+ complex. |
| A1, primary auditory cortex; IPL, inferior parietal lobule; IPS, intraparietal sulcus; LOC, lateral occipital cortex; MDN, medial dorsal nucleus; MOG, middle occipital gyrus; MTG, middle temporal gyrus; PPC, posterior parietal cortex; SMG, supramarginal gyrus; SOG, superior occipital gyrus; SPL, superior parietal lobe; V1, primary visual cortex; | | | | | | |

**Supplementary references**

ANUROVA, I., CARLSON, S. & RAUSCHECKER, J. P. 2019. Overlapping Anatomical Networks Convey Cross-Modal Suppression in the Sighted and Coactivation of "Visual" and Auditory Cortex in the Blind. *Cereb Cortex,* 29**,** 4863-4876.

ARNOTT, S. R., THALER, L., MILNE, J. L., KISH, D. & GOODALE, M. A. 2013. Shape-specific activation of occipital cortex in an early blind echolocation expert. *Neuropsychologia,* 51**,** 938-49.

COLLIGNON, O., DORMAL, G., ALBOUY, G., VANDEWALLE, G., VOSS, P., PHILLIPS, C. & LEPORE, F. 2013. Impact of blindness onset on the functional organization and the connectivity of the occipital cortex. *Brain,* 136**,** 2769-83.

DE VOLDER, A. G., CATALAN-AHUMADA, M., ROBERT, A., BOL, A., LABAR, D., COPPENS, A., MICHEL, C. & VERAART, C. 1999. Changes in occipital cortex activity in early blind humans using a sensory substitution device. *Brain Res,* 826**,** 128-34.

GARG, A., SCHWARTZ, D. & STEVENS, A. A. 2007. Orienting auditory spatial attention engages frontal eye fields and medial occipital cortex in congenitally blind humans. *Neuropsychologia,* 45**,** 2307-21.

GOYAL, M. S., HANSEN, P. J. & BLAKEMORE, C. B. 2006. Tactile perception recruits functionally related visual areas in the late-blind. *NeuroReport,* 17.

HOFSTETTER, S., ZUIDERBAAN, W., HEIMLER, B., DUMOULIN, S. O. & AMEDI, A. 2021. Topographic maps and neural tuning for sensory substitution dimensions learned in adulthood in a congenital blind subject. *Neuroimage,* 235**,** 118029.

HUBER, E., JIANG, F. & FINE, I. 2019. Responses in area hMT+ reflect tuning for both auditory frequency and motion after blindness early in life. *Proc Natl Acad Sci U S A,* 116**,** 10081-10086.

JIANG, F., STECKER, G. C., BOYNTON, G. M. & FINE, I. 2016. Early Blindness Results in Developmental Plasticity for Auditory Motion Processing within Auditory and Occipital Cortex. *Frontiers in Human Neuroscience,* 10.

JIANG, F., STECKER, G. C. & FINE, I. 2014. Auditory motion processing after early blindness. *J Vis,* 14**,** 4.

LEWIS, L. B., SAENZ, M. & FINE, I. 2010. Mechanisms of cross-modal plasticity in early-blind subjects. *J Neurophysiol,* 104**,** 2995-3008.

LIKOVA, L. 2012. The Spatiotopic 'Visual' Cortex of the Blind. *HUMAN VISION AND ELECTRONIC IMAGING XVII.*

LIKOVA, L. T. 2010. Drawing in the blind and the sighted as a probe of cortical reorganization. *HUMAN VISION AND ELECTRONIC IMAGING XV.*

MAIDENBAUM, S., CHEBAT, D.-R. & AMEDI, A. 2018. Human Navigation Without and With Vision - the Role of Visual Experience and Visual Regions. *bioRxiv***,** 480558.

NORMAN, L. J. & THALER, L. 2019. Retinotopic-like maps of spatial sound in primary 'visual' cortex of blind human echolocators. *Proc Biol Sci,* 286**,** 20191910.

POIRIER, C., COLLIGNON, O., SCHEIBER, C., RENIER, L., VANLIERDE, A., TRANDUY, D., VERAART, C. & DE VOLDER, A. G. 2006. Auditory motion perception activates visual motion areas in early blind subjects. *Neuroimage,* 31**,** 279-85.

STRNAD, L., PEELEN, M. V., BEDNY, M. & CARAMAZZA, A. 2013. Multivoxel Pattern Analysis Reveals Auditory Motion Information in MT+ of Both Congenitally Blind and Sighted Individuals. *PLOS ONE,* 8**,** e63198.

THALER, L., ARNOTT, S. R. & GOODALE, M. A. 2011. Neural correlates of natural human echolocation in early and late blind echolocation experts. *PLoS ONE,* 6(5) (no pagination).

THALER, L., MILNE, J. L., ARNOTT, S. R., KISH, D. & GOODALE, M. A. 2014. Neural correlates of motion processing through echolocation, source hearing, and vision in blind echolocation experts and sighted echolocation novices. *J Neurophysiol,* 111**,** 112-27.

VAN DER HEIJDEN, K., FORMISANO, E., VALENTE, G., ZHAN, M., KUPERS, R. & DE GELDER, B. 2020. Reorganization of Sound Location Processing in the Auditory Cortex of Blind Humans. *Cereb Cortex,* 30**,** 1103-1116.

WALLMEIER, L., KISH, D., WIEGREBE, L. & FLANAGIN, V. L. 2015. Aural localization of silent objects by active human biosonar: Neural representations of virtual echo-acoustic space. *European Journal of Neuroscience,* 41(5)**,** 533-545.

WOLBERS, T., ZAHORIK, P. & GIUDICE, N. A. 2011. Decoding the direction of auditory motion in blind humans. *Neuroimage,* 56**,** 681-7.
